# Collateral Damage Intended—Cancer-Associated Fibroblasts and Vasculature Are Potential Targets in Cancer Therapy

**DOI:** 10.3390/ijms18112355

**Published:** 2017-11-07

**Authors:** Ana Cavaco, Maryam Rezaei, Stephan Niland, Johannes A. Eble

**Affiliations:** Institute of Physiological Chemistry and Pathobiochemistry, Münster University Hospital, 48149 Münster, Germany; acmcavaco@gmail.com (A.C.); mrezaei@uni-muenster.de (M.R.)

**Keywords:** abnormal tumor vasculature, anti-angiogenesis, cancer-associated fibroblasts, endothelial cell–tumor cell interaction, targeted tumor therapy, tumor neovascularization, tumor metabolism, tumor stroma, tumor vessel disruption, vasculogenic mimicry

## Abstract

After oncogenic transformation, tumor cells rewire their metabolism to obtain sufficient energy and biochemical building blocks for cell proliferation, even under hypoxic conditions. Glucose and glutamine become their major limiting nutritional demands. Instead of being autonomous, tumor cells change their immediate environment not only by their metabolites but also by mediators, such as juxtacrine cell contacts, chemokines and other cytokines. Thus, the tumor cells shape their microenvironment as well as induce resident cells, such as fibroblasts and endothelial cells (ECs), to support them. Fibroblasts differentiate into cancer-associated fibroblasts (CAFs), which produce a qualitatively and quantitatively different extracellular matrix (ECM). By their contractile power, they exert tensile forces onto this ECM, leading to increased intratumoral pressure. Moreover, along with enhanced cross-linkage of the ECM components, CAFs thus stiffen the ECM. Attracted by tumor cell- and CAF-secreted vascular endothelial growth factor (VEGF), ECs sprout from pre-existing blood vessels during tumor-induced angiogenesis. Tumor vessels are distinct from EC-lined vessels, because tumor cells integrate into the endothelium or even mimic and replace it in vasculogenic mimicry (VM) vessels. Not only the VM vessels but also the characteristically malformed EC-lined tumor vessels are typical for tumor tissue and may represent promising targets in cancer therapy.

## 1. Introduction

In the last few decades, tumor therapy has made appreciable progress. In addition to surgical intervention, radio- and chemotherapy have significantly increased survival of tumor patients. Most recently, immunotherapy directed against immune checkpoint inhibitors has been improved and advanced to first line therapy for different cancers [1,2].

While the oncogenically transformed tumor cell has been and will continue to be the focus of cancer therapy, an increasing number of publications in recent years has also shed light on cells in the vicinity of tumor cells and their role in tumor progression. Stromal fibroblasts, endothelial cells (ECs) and immune cells belong to this cellular environment. They are not unaffected bystanders, but their behavior changes in response to neighboring tumor cells. Thus, they may support growth and progression of cancer cells which eventually subvert the resident cells. This review highlights metabolic alterations and intercellular communication of tumor cells and their neighboring stromal fibroblasts and ECs. In addition, immune cells, such as macrophages, granulocytes, and leukocytes, are affected in a solid tumor and in turn affect tumor growth. These immunological aspects have been excellently reviewed elsewhere [3] and will not be covered here. This review focuses on fibrotic and vascular phenomena within growing solid tumor tissue.

## 2. Setting the Stage: Cancer Cells Determine the Tumor Microenvironment via Metabolites and Cytokines, via Cell–Matrix and Cell–Cell Contacts

### 2.1. Metabolic Reprogramming of Cancer Cells

**Figure 1:**
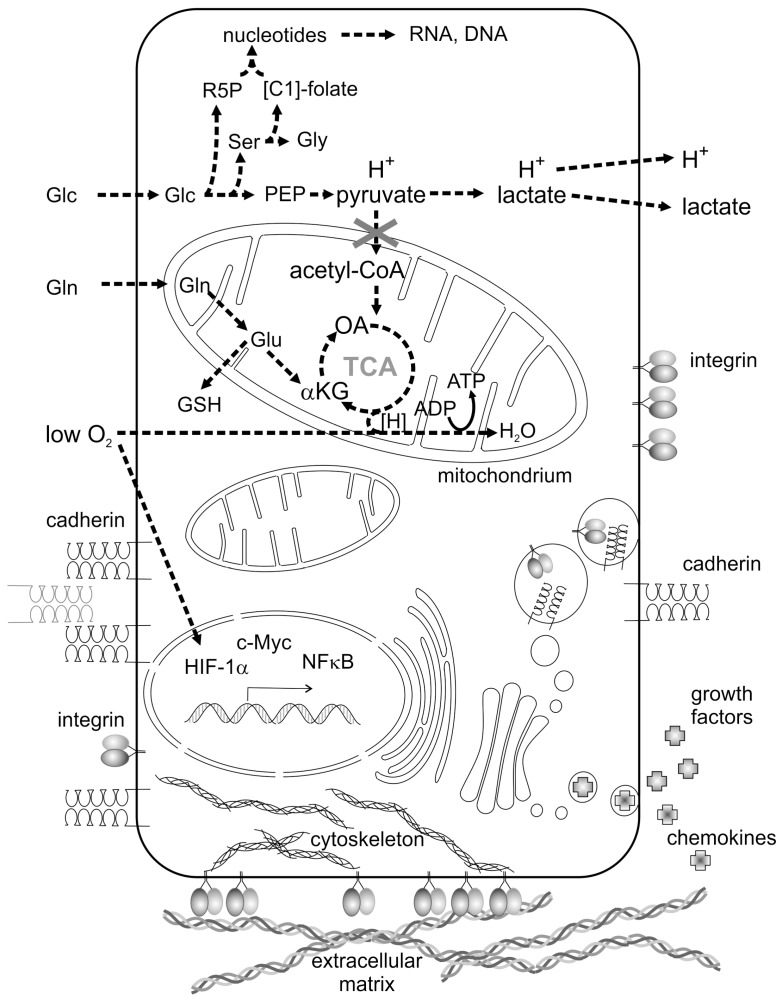
Metabolic reprogramming and an altered intercellular communication are hallmarks of cancer cells. Enhanced demands of glucose (Glc) and glutamine (Gln) as well as low supply of oxygen are characteristic features of cancer cell metabolism. They activate distinct transcription factors, such as hypoxia-inducible factor 1α (HIF-1α), cellular Myelocytomatose (c-Myc) and nuclear factor κ-light-chain-enhancer of activated B cells (NFκB), and upregulate expression of glycolytic and glutaminolytic key enzymes. Aerobic glycolysis leads to a high lactate concentration and a low pH of the tumor microenvironment (TME). Glycolytic metabolites stimulate the pentose phosphate pathway to produce ribose-5-phosphate (R5P) and the production of the amino acids serine (Ser) and glycine (Gly), thereby filling the tetrahydrofolate pool of C1-groups ([C1]-folate). The tricarboxylic acid (TCA) cycle is fueled by glutamine (Gln) via glutamate (Glu) and α-ketoglutarate (αKG). Glutamate is also converted to glutathione (GSH), an intracellular redox buffer. Metabolites, phosphoenolpyruvate and oxaloacetate are abbreviated to PEP and OA, respectively. Membrane-bound cell adhesion molecules (e.g., integrins) and cell–cell contact molecules (e.g., cadherin), as well as secreted and soluble growth factors and chemokines are other key communicator molecules between cancer cells and their neighboring stromal cells. Cell adhesion molecules bind to the extracellular matrix (ECM) and sense its rigidity and mechanical forces.

Proliferating tumor cells lack oxygen due to a malfunction or even absence of a proper tumor vasculature. Lack of oxygen strongly contributes to a reprogramming of cancer cell metabolism and is typical of the tumor microenvironment (TME) [4]. Driven by the oxygen-dependent hypoxia-inducible transcription factor-1α (HIF-1α) [4] and by the transcription factor cellular Myelocytomatose (c-Myc) [5], expression of key enzymes which regulate fundamental metabolic pathways is controlled in an orchestrated and cancer cell-specific way [6,7,8]. Glycolysis and glutaminolysis are the most prominently activated pathways in cancer cells (Figure 1) which, together with hypoxia, belong to the metabolic hallmarks of cancer [9]. The prime carbon and energy source of proliferating tumor cells is glucose (Glc in Figure 1), which, after uptake by glucose transporter tye 2 (GLUT2), is utilized via glycolysis. Glycolytic key enzymes, such as hexokinase 2 and the pyruvate kinase isoform M2 (PK-M2), are upregulated [10]. Moreover, PK-M2 forms a less active dimer instead of the highly active tetramer found in normal cells [11,12]. Only high concentrations of fructose-1,6-bisphosphate triggers the formation of the enzymatically active tetramer of PK-M2 [11,12]. Reduced pyruvate kinase activity results in accumulation of upstream metabolites [7,8,13], such as phosphoenolpyruvate (PEP in Figure 1), prompts the synthesis of the amino acids serine (Ser) and glycine (Gly) and stimulates the flow of metabolites into the pentose phosphate pathway, which yields nicotinamide adenine dinucleotide phosphate (NADPH + H^+^) and ribose-5-phosphate (R5P), the building block for nucleotides, RNA and DNA. Folate-bound C1 bodies (methylene, hydroxymethyl, formyl groups) for purine and pyrimidine synthesis are provided by the conversion of serine to glycine [6,12].

Instead of being transported into mitochondria, the end product of glycolysis, pyruvate, is cytosolically reduced to lactic acid, which dissociates into lactate and protons, and both are transported out of the cells. This explains the tumor-characteristic increase of extracellular lactate and the acidification of the tumor environment. Almost a century ago, Otto Warburg discovered that tumor cells prominently use glycolysis, even if sufficient oxygen is provided [14]. The lactic acid produced by aerobic glycolysis fails to feed the mitochondrial tricarboxylic acid (TCA) cycle [15]. To fuel the TCA cycle, glutamine (Gln in Figure 1) becomes another carbon source of the cancer cell metabolism. Glutamine utilization is mainly regulated by the glutaminase transporter and by mitochondrial glutaminase-1 in a c-Myc-dependent manner [16]. Its product, glutamate, not only replenishes the TCA cycle, but also serves as starting material for glutathione (GSH) synthesis in the cytosol. As GSH is part of the predominant intracellular redox buffer, the increased glutamine demand of tumor cells also affects redox homeostasis. The end product of glutaminolysis, α-KG, not only fuels the TCA cycle but can be converted by mutated isocitrate dehydrogenase isoforms to 2-hydroxyglutarate, whose concentration is elevated in several brain tumors [17].

This characteristic reprogramming of the metabolism allows to identify and to target tumor cells for diagnosis and therapy of cancer patients. Increased uptake and utilization of glucose and glutamine by cancer cells is diagnostically exploited by using 2-deoxy-2-(^18^F)fluoro-D-glucose (^18^FDG) in positron emission tomography-computed tomography (PET-CT) and labeled glutamine derivatives [16]. Several transport proteins for glucose and glutamine, as well as key enzymes of the aberrantly activated glycolysis and glutaminolysis, have been identified as therapeutic targets in cancer therapy, such as GLUT2, hexokinase-2, pyruvate kinase type M2 [8,10], glutaminase-1 and glutamate dehydrogenase [16,18].

The metabolic reprogramming of tumor cells and secretion of metabolites also contribute to communication with stromal cells. In some cancer types, cancer-associated fibroblasts (CAFs) and cancer cells seem to establish a symbiotic relationship regarding their energy metabolism [19,20,21]. Lactate, produced and secreted by cancer cells is taken up by CAFs and utilized as an energy source for their pro-tumorigenic functions [22]. Conversely, cancer cells release reactive oxygen species (ROS) that induce aerobic glycolysis in CAFs, which leads to secretion of additional lactate and pyruvate. They may provide metabolic energy for cancer cells [22,23]. The direction in which the lactate/pyruvate flows depends on the conditions of the TME [19]. Caused by excess lactic acid production, the pH drop likely contributes to the acquisition of drug resistance in tumor cells [24]. Likewise, ECs near tumor cells adjust their metabolism and alter the glycolytic metabolism, a property that has recently been highlighted to be a potential therapeutic approach [25,26].

### 2.2. Cohesion, Adhesion and Soluble Mediators in the Communication between Tumor Cells

Cell–cell contacts (cohesion) between layer-forming epithelial and ECs are mediated by cadherins and other cell–cell contact molecules. Epithelial cell-derived carcinoma cells typically express E-cadherin, while VE-cadherin is the principal cadherin of ECs. Cadherins are transmembrane proteins consisting of five extracellular IgG-folds, a transmembrane part and a cytoplasmic tail, the latter of which is anchored via α-, β-, and γ-catenins to the actin cytoskeleton [27]. Two cadherin molecules of one cell form a homodimer which interacts with a cadherin homodimer of the same type on a neighboring cell in a Ca^2+^-ion dependent manner, thus mediating cell-type specific cohesion and ruling out interactions with cells of other tissues which bear other cadherin types (Figure 1). While E-cadherin-expressing carcinoma cells cohere, loss of cadherin expression or function promotes contact loss to neighboring tumor cells. Thus, a carcinoma cell can disseminate from a tumor cell cluster, a hallmark of malignancy. A detached carcinoma cell changes its cellular morphology and increases its migratory potential, a process called epithelial–mesenchymal transition (EMT). EMT correlates with tumor cell scattering and metastasis [28]. E-Cadherin surface exposure is regulated at the transcriptional level by the key transcription factors, Snail family transcriptional repressor 1 (SNAI1) and TWIST1, and by epigenetic factors, such as DNA-hypermethylation, as well as by endocytosis and subsequent degradation [28]. Moreover, growth factor receptors, such as Epidermal growth factor receptor (EGFR) and hepatocyte growth factor receptor (HGFR, c-Met), may activate Src, which triggers phosphorylation and endocytosis of E-cadherin, leading to dissemination of tumor cells from the tumor mass. Even if not completely abolished, reduced E-cadherin levels have been observed in subgroups of carcinoma cells, which migrate collectively. Downregulation of E-cadherin during EMT may be accompanied by the upregulation of mesenchymal cadherins, such as N-cadherin and cadherin-11, which allow new interactions of tumor cells with stromal fibroblasts. Moreover, by expressing VE-cadherin, tumor cells may also mimic ECs and thus are able to establish unconventional interactions with ECs. Such heterotypic cohesion events may enable tumor cells to contact with stromal fibroblasts and ECs directly via cell–cell contacts [28].

Adhesion is the interaction of cells with their extracellular matrix (ECM). As a three-dimensional interstitial fibrillar meshwork, the ECM scaffolds the stromal tissue and as a two-dimensional basement membrane (BM), it supports epithelial or endothelial tissue layers. Integrins, heterodimeric transmembrane proteins consisting of an α subunit and a subgroup-determining β-subunit, are the corresponding adhesion receptors on adherent cells (Figure 1). Integrins with a β1, β3, and β4 subunit bind via their ectodomains to ECM proteins, which trigger integrin clustering and subsequent signaling. Lacking a kinase domain, integrins interact with several adaptor, signaling, and cytoskeletal proteins via their cytoplasmic domains, thereby transducing both environmental cues and mechanical forces between the ECM and the cytoskeleton [29,30]. It is of special interest that tumor cells generate mechanical forces via actin-associated motor proteins, such as myosin II. These intracellular forces are transmitted to the ECM network via integrins and build up tension in it. This mechanical tension is another key parameter which determines the TME and is sensed by resident cells, e.g., fibroblasts. Diagnostically, the BM plays a pivotal role in tumor metastasis. It is a sheet-like matrix structure containing characteristic proteins, such as type IV collagen, laminins, nidogens, and the principal BM proteoglycan perlecan. In addition to its physiological functions as morphogen, the BM acts as a cell barrier. Physiologically, it can only be penetrated by leukocytes during immune surveillance of tissues. Pathologically, oncogenically transformed tumor cells are able to breach the BM due to their altered integrin repertoire and expression of ECM degrading matrix metalloproteinases (MMPs), and thus they are considered malignant [31]. Breaching the BM defines malignancy and is another hallmark of cancer [9].

Growth factors and chemokines are other means of communication between tumor cells and within the TME (Figure 1). These soluble signaling molecules are produced by tumor cells or by resident cells. As a consequence of the TME, tumor cells may produce growth factors, such as Hepatocyte growth factor (HGF), Fibroblast growth factors (FGFs), Transforming growth factor β isoforms (TGFβs), Vascular endothelial growth factors isoforms(VEGFs), and cytokines, such as Receptor activator of nuclear factor kappa-B ligand (RANKL) and other members of the Tumor necrosis factor α (TNFα) superfamily [32,33]. This cocktail of growth factors and cytokines also contributes to the specific TME. The growth factors act in an autocrine and/or paracrine manner on tumor cells and/or resident cells, and stimulate their proliferation. Tumor cells alter the expression and activity of secreted cytokines as well as of various cytokine receptors. This alters their responsiveness to such factors. Several mutations in growth factor receptors, such as EGFR, hepatocyte growth factor receptor (cMET), and Fibroblast growth factor receptor isoforms (FGFRs), have been described to initiate uncontrolled cell proliferation of transformed cells [34,35]. Secreted by cancer cells, transforming growth factor-β (TGFβ) is a key driver in the differentiation of fibroblasts to Cancer-associated fibroblasts (CAFs). VEGF-A produced by tumor cells, under hypoxic conditions, attracts ECs to the tumor cell mass resulting in tumor-induced angiogenesis. Conversely, growth factors and cytokines produced by the resident cells may affect the cancer cells and may induce them to change their repertoire of integrins and cadherins [36,37,38]. For example, after stimulation by HGF, cMet triggers the internalization and subsequent degradation of E-cadherin in carcinoma cells, resulting in EMT and tumor cell dissemination [28].

In all body fluids, extracellular membrane vesicles (EVs) of different size, such as exosomes, microparticles or microvesicles, and apoptotic bodies, contain numerous signaling molecules dependent on their cellular origin. They are released from sender cells to be taken up by target cells. In this way, they convey intercellular signals in autocrine, paracrine, and even endocrine manners [39,40,41]. Thus, they crucially mediate intratumoral signaling, tumor progression, metastasis, and chemotherapy resistance. Exosomes with a diameter of 30–100 nm generally contain membrane fusion proteins (e.g., tetraspanins, lactadherin, and integrins), cytoskeletal proteins (e.g., actin and tubulin), membrane trafficking proteins (e.g., Rab proteins, ADP ribosylation factor (ARF) GTPases, and annexins), cytoplasmic enzymes (e.g., Glyceraldehyde 3-phosphate dehydrogenase (GAPDH), peroxidases, pyruvate kinases, and lactate dehydrogenase) and, signal transduction proteins (e.g., protein kinases and heterotrimeric G-proteins) [42,43].

Tumor cells communicate with neighboring resident cells via local metabolic parameters, such as lactic acid-mediated acidosis, low oxygen supply and increased ROS levels. Furthermore, secreted mediators, such as growth factors and chemokines, and the composition and mechanical tension of the ECM and integrin-mediated cell–matrix contacts, as well as cadherin-mediated cell–cell contacts are other means of communication in the TME (Figure 1). These factors determine the TME, in which the resident cells change their metabolism and behavior in support of the tumor cell. This niche supports cancer progression and can be compared to the “soil” in which, according to Stephen Paget’s “seed and soil” theory (1889) [44], cancer cells thrive or metastasizing cells settle. The tumor cells prepare this “soil” either directly or by making neighboring cells, such as fibroblasts and ECs, change the “soil” in favor of the tumor.

## 3. Stromal Fibroblasts, the Immediate Neighbors of Tumor Cells

The TME constitutes a very complex niche, with extreme importance for the maintenance and progression of the tumor cells [45]. It consists of two components: cells and the ECM. The tumor stroma, or “reactive stroma” comprises three important cell groups [19,46]: CAFs (described in more detail in this section), angiogenic vascular cells (discussed in the next section) and infiltrating immune cells [3]. The pro-tumorigenic TME is characterized by an increased deposition and an altered composition of the ECM, by higher microvessel density, and by the activation of cancer-recruited stromal cells [46]. However, the TME differs between tumors, with diverse tumor stroma composition and different portions and activation states of stromal cells and it may alter during tumor progression, due to the evolving environmental conditions and oncogenic signals from growing tumors [47]. Differences in the TME are also observed within the same tumor, with disparities between the invasive edge and the tumor core, in line with the metabolic alterations, such as the availability of oxygen and nutrients [47]. Additionally, the presence of different cell types producing specific growth factors influences the tumor cells differently [48]. Finally, mechanical aspects of the tumor stroma, such as stiffness of the ECM and interstitial fluid pressure, play a crucial role in the TME [46,49]. As complex and diverse as it is, the TME dictates the fate of the tumor by providing survival and expansion signals, by setting the selection criteria of mutant subclones and by creating tumor cell heterogeneity, thereby posing an enormous challenge in cancer therapy [48].

### 3.1. CAFs Are Crucial for the Maintenance of a Pro-Tumorigenic TME

CAFs are the most prominent cell type in the tumor stromal compartment. They are crucial in forming and maintaining a pro-tumorigenic niche. Their presence in the tumor tissue has been associated with a poor prognosis in many cancer types as, e.g., gastric [50], colon [51], breast [52], and pancreatic cancers [53]. CAFs have been described as myofibroblasts, resembling the activated fibroblasts in wound healing. In some aspects, the tumor stroma is similar to granulation tissue, since the main cellular components are fibroblasts, together with immune, inflammatory and ECs [54]. Furthermore, in both tumor progression and wound healing, more ECM is deposited and cross-linked. As a consequence of this, the ECM scaffold is remarkably stiffened. In addition, more soluble cytokines, such as TGFβ1, are tethered to the ECM scaffold [54,55]. Dvorak even defined a tumor as “a wound that never heals” [54].

Normally, the ECM is sparsely populated by undifferentiated spindle-shaped fibroblasts [56]. When tissue injury takes place, these fibroblasts are activated. They start to express high levels of α-smooth muscle actin (αSMA), gain a stellate shape and produce more ECM [56]. Differentiating into myofibroblasts, they acquire contractile properties to close the wound. Moreover, they take on a secretory, migratory, and proliferatory phenotype. This further enhances activation and recruitment to the damaged tissue [56,57]. Once wound healing is accomplished, these cells revert to their normal phenotype or undergo apoptosis [56,58]. In a neoplastic lesion, this reversion or apoptosis does not happen. Instead, their proliferation, secretion of paracrine and autocrine cytokines [59], and ECM production and remodeling are enhanced [60]. Among the cytokines, TGFβ1, monocyte chemotactic protein (MCP1), platelet-derived growth factor (PDGF), and FGF, as well as secreted proteases have been implicated in CAFs activation [61,62]. Cancer cell-derived exosomes containing TGFβ and betaglycan have been reported to induce differentiation of fibroblasts to myofibroblasts by SMAD signaling and upregulation of basic FGF (bFGF, FGF2) production and α-smooth muscle actin expression [63]. While normal fibroblasts were reported to suppress tumor formation [64], CAFs emerge in the tumor as promoters of a pro-tumorigenic TME and thus lay an indispensable foundation for cancer progression. What is the origin of the CAFs present in the TME? There are several and controversial hypotheses about possible precursor cells and about different stimuli which ultimately induce formerly tumor-suppressing fibroblasts to express miscellaneous other marker proteins and change their phenotype into that of pro-tumorigenic CAFs. In accordance to these hypotheses is a description of CAFs as a heterogeneous cell population with numerous and different functions in the tumor. This heterogeneity complicates their investigation.

Specific markers for CAFs have not yet been identified, but diverse proteins are altered upon differentiation of fibroblasts into CAFs. αSMA, a component of cytoskeletal stress fibers, was one of the first proteins to be described as a marker for myofibroblasts in both fibrotic tissue and cancer [65]. In addition, a filament-associated, calcium-binding protein called fibroblast-specific protein 1 (FSP1) was typically expressed de novo in activated fibroblasts [66]. Platelet derived growth factor receptor-β (PDGFRβ) and NG2 chondroitin sulfate proteoglycan (NG2) were found in some populations of pancreatic CAFs, in co-localization with αSMA and FSP1, albeit in different percentages. This may indicate different subpopulations of CAFs [67]. Fibroblast activation protein (FAP) is another marker, originally described as a cell surface glycoprotein of reactive stromal fibroblasts [68]. ECM protein tenascin C was also described to be a typical secretion product and hence potential marker of CAFs [69]. The lack of a universal CAFs marker is likely to be due to the diversity of CAFs. Depending on the tumor type and organ in which they differentiate, diverse CAF populations exist which possess different characteristics [51]. Herrera et al. showed that different subpopulations of colon CAFs, obtained from different patients, had distinct promigratory effects on colon cancer cells [51]. The diversity of tumor CAFs may be rooted in their origin [51]. CAFs can originate from several cell types, such as normal fibroblasts, myofibroblasts, adipocytes, smooth muscle cells, or bone marrow-derived progenitor cells [70,71]. Moreover, the differentiation pattern of CAFs may depend on environmental cues provided by different components of the TME, such as the ECM and the cytokine mixture. Local fibroblasts from the stroma where the neoplastic lesion develops can differentiate into CAF, as a result of stimulation by cytokines of the PDGF or TGFβ family produced by the cancer cells, macrophages and other stromal cells [72,73]. CAFs may also originate from ECs in a process called endothelial to mesenchymal transition, which ECs undergo when submitted to fibrotic conditions, e.g., under the influence of TGFβ1 [74]. By using two different tumor mouse models (pancreatic neuroendocrine tumor and melanoma), Zeisberg et al. demonstrated, that ECs acquire a mesenchymal phenotype and express markers such as FSP1, and to a smaller extent, αSMA [74]. This study showed that ECs are a possible source for CAFs in the microenvironment of angiogenic tumors.

Various functions are attributed to CAFs. Due to their acquired secretory phenotype, they play a central role in processes such as EMT, angiogenesis and immune cell recruitment. CAFs secrete TGFβ1 and thus induce EMT in many carcinomas by TGFβ1-mediated loss of adherens junctions and by increased motility of cancer cells which results in enhanced invasion and metastasis abilities [59,75]. Moreover, some of the first studies on the role of stromal cells in tumor angiogenesis used transgenic mice expressing green fluorescent protein (GFP) under the control of the vascular endothelial growth factor (VEGF) promoter. In spontaneous mammary tumors, as in wounds, the predominant GFP-positive cells were fibroblasts [76]. CAFs also have been reported to promote angiogenesis by different mechanisms: mouse cervical CAFs produce pro-angiogenic fibroblast growth factors FGF-2 and FGF-7 and, consequently interception of FGF impairs angiogenesis [77]. Another CAF-related mechanism to stimulate angiogenesis is to recruit endothelial progenitor cells (EPCs) into the carcinoma site by secretion of stromal cell-derived factor 1 (SDF-1), also known as C-X-C motif chemokine 12 (CXCL12) [78]. Moreover, Orimo et al. described that the interaction of CAF-secreted CXCL12 with its receptor C-X-C chemokine receptor type 4 (CXCR-4 (CXCR4), expressed by carcinoma cells, results in enhanced tumor growth [78]. This chemokine is also associated with an inflammatory response by recruiting leukocytes into the tumor stroma, where they contribute to angiogenesis by producing angiogenic factors, by remodeling the ECM via stimulated secretion of MMP-9, and by direct differentiation into ECs [79,80,81]. Moreover, immunosuppressive CAFs at the invasive front of a tumor interfere with dendritic cell differentiation [47]. CAFs also modulate the immune response by secreting cytokines and chemokines, such as interleukin-1 and MCP1, respectively [62]. In addition to cytokines, CAFs secrete exosomes, containing soluble factors that promote breast cancer cell migration. Such exosomes are yet another means of communication between cancer cells and stroma cells, but also between primary and secondary sites of a tumor [82].

### 3.2. ECM Is a Means of Communication in the TME and Signals via Distinct Parameters: Qualitative and Quantitative Composition, Cross-Linkage of Supramolecular Structures, Tensional Status and Degradation

The ECM forming the extracellular scaffold for fibroblasts is the characteristic component of connective tissue. Its border, the BM, forms the foundation to which cells of all other tissues, such as epithelial and ECs, muscle cells, neurons and adipocytes, are anchored. However, during carcinogenesis, the ECM is remodeled. This is mainly done by stromal cells, such as CAFs [83]. Moreover, breaching of the BM by tumor cells is a hallmark of malignancy.

The constitution of the ECM in different tumor types is highly heterogeneous. In addition, within the same tumor, differences can be noted, as ECM deposition may change depending on tumor staging [84]. Different types of collagens, laminins, proteoglycans, glycosaminoglycans, fibronectin, and vitronectin are among the most abundantly expressed ECM proteins in cancer stroma. They are deposited and remodeled by stromal cells, such as CAFs. The ECM in the TME is also functionally diverse, and the multiple interactions between the different constituents increase this diversity.

As major components of the BM, laminins are crucial in tumor angiogenesis and metastasis [85,86]. Usually laminin α4 chain is overexpressed in breast cancer and promotes cell detachment in vitro, and in vivo it stimulates tumor re-initiation in multiple organs, and disseminated metastatic cell proliferation [87].

Expression of fibronectin is upregulated in CAFs at metastatic sites, e.g., in the lung, and serves as a docking site for the hematopoietic progenitor cells and invading tumor cells [47]. Being part of the TME scaffold of aggressive tumors, it comes in two different splice variants which differ in the presence of the extra-domains (ED) A or B, called EDA and EDB [88,89,90]. Bordeleau et al. described the alternative splicing as an adaptation of the cells to their microenvironment [91]. The increased production of the EDB fibronectin isoform by ECs correlates with ECM stiffness [91]. Matrix stiffness-regulated splicing depends on the activation of various splice factors, on intracellular Rho/Rho-associated protein kinase (ROCK)-mediated contractility and on PI3K-AKT signaling [91]. Regulation of alternative splicing by ECM stiffness is likely to occur in other cell types, too [91]. In contrast, the alternatively spliced EDA fibronectin variant is deposited in regions of active fibrosis, e.g., in idiopathic pulmonary fibrosis [92,93]. In this context, EDA fibronectin plays a role in TGFβ-dependent differentiation of fibroblasts into myofibroblasts via autocrine/paracrine feedback loops and in metastasis, while EDB fibronectin is likely involved in EC proliferation and vascular morphogenesis, tumorigenesis and EMT [88,94].

Tenascin-C and periostin are matricellular proteins produced by CAFs. They collaboratively contribute to lung metastasis, in a process involving Wingless-related integration site (Wnt) and Notch signaling pathways [95]. Periostin recruits Wnt ligands and presents them to stem-like metastasis-initiating cells [96]. On the other hand, tenascin-C, produced by both CAFs and tumor cells, activates Wnt and Notch pathways, supporting the fitness of metastasis-initiating breast cancer cells and their “seeding” at the metastatic site [97]. Moreover, periostin also contributes to proper assembly and homeostasis of collagen. In addition, its deposition enables tenascin-C to bind to other ECM molecules such as collagen-I and fibronectin [98,99]. Tenascin-W, the fourth and newest member of the tenascin family, was discovered ten years ago. It is expressed in activated tumor stroma, facilitating tumorigenesis by supporting the migratory behavior of breast cancer cells [100]. Both tenascin-C and -W can be expressed in tumor stroma usually at similar percentages, being most likely produced by CAFs [101]. However, they do not necessarily coexist in a tumor, likely due to independent modulation mechanisms [101]. For example, tenascin-W is enriched in low-grade cancers, while tenascin-C expression is found irrespective of the tumor grade [100]. In addition, in colon cancer, tenascin-W, in contrast to tenascin-C, is ectopically expressed in tumor tissue and is considered as cancer biomarker of unfavorable disease progression, since it is not detectable in healthy colon stroma [102]. Moreover, tenascin-W is present in the stroma of mouse mammary tumor models developing metastasis, whereas tenascin-C is absent from both non-metastatic tumors and normal mammary tissue [103].

A fibrotic overexpression of collagenous ECM components contributes to a desmoplastic TME [83]. The mechanical robustness and stiffness of the ECM is strongly increased through inter- and intramolecular cross-linkages of fibrous collagen and elastin. They are catalyzed by members of the lysyl oxidase (LOX) gene family, such as lysyl oxidase-like protein-1 (LOXL1). The expression of this amine oxidase seems to correlate with increased tumor malignancy, since it is expressed in metastatic but not in non-metastatic cell lines [104,105]. Other experimental observations point out that LOXL1-expressing tumors are highly fibrotic and surrounded by many dense collagen fibers, [104]. Inhibition of LOX-dependent collagen crosslinking decreases tissue desmoplasia, tumor incidence and growth, and reduces mechanotransduction in the mammary epithelium [49].

The mechanical forces that increase ECM stiffening and intratumoral pressure are generated intracellularly by cytoskeletal motor proteins and transmitted via transmembrane integrins to ECM proteins such as collagens and laminins. The integrin repertoire of tumors alters during cancerogenesis [106]. Integrins are both mechanotransducers of tensile force and also elicit intracellular signaling pathways. Thereby, they regulate cell differentiation and fate [106]. Tumor cells express, e.g., β4 integrins which endow them with resistance to apoptosis [107]. In addition, β1-integrin expression has been described as critical for tumorigenesis initiation and for maintaining the proliferative capacity of late-stage tumor cells [108].

TGFβ stimulates CAFs by autocrine signaling to produce and deposit more collagens I and III and fibronectin, which then promote cell adhesion and strengthen mechanical signaling between CAFs and tumor cells [83]. Noteworthy, the ECM can also tether and store growth factors, e.g., latent TGFβ1 [109]. Integrin-mediated ECM contraction by CAFs releases TGFβ1 from ECM fibers under tension, especially in a fibrotic and stiffened matrix, and protease-independently activates TGFβ1 [109]. Excess production, remodeling, stiffening of the ECM and CAF differentiation mutually promote each other, resulting in increased release of TGFβ1 into the TME. Such self-sustaining growth signals promote cell activation, proliferation, and EMT, thereby reinforcing tumor progression [109].

**Figure 2:**
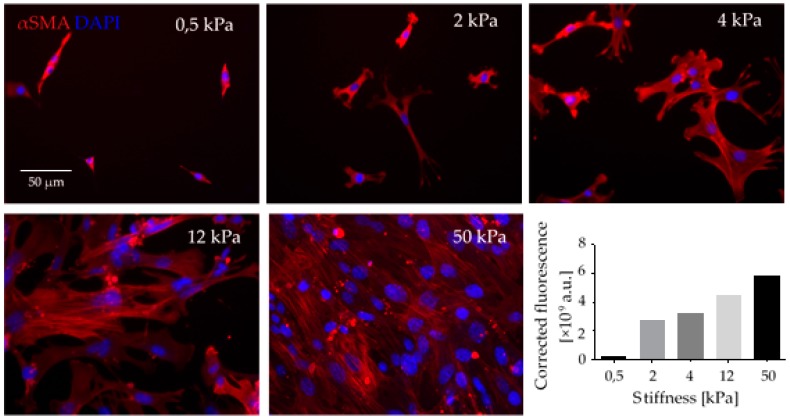
Mechanical stiffness of ECM is a crucial factor in CAF differentiation. Fibroblasts seeded in collagen-I coated polyacrylamide gels of defined stiffness (elastic modulus is given in kPa) exhibited increased adhesion and increased formation of α-Smooth muscle actin (αSMA)-rich stress fibers (red fluorescence). αSMA immunostaining was quantified as total corrected fluorescence. This experiment reflects in vivo conditions, where the stiff scaffold of desmoplastic ECM contributes to CAF differentiation, together with soluble factors such as TGFβ that are stored bound to ECM fibers and released when CAFs exert force on those fibers. Upon differentiation, CAFs change their morphology and express different biomarkers, such as αSMA stress fibers (red fluorescence). CAFs proliferate at higher rate, exhibit a secretory phenotype and enhanced contractibility; thus, they play an essential role in forming the TME. Scale Bar = 50 μm.

Matrix stiffening and increased tensile forces modulate the cytoskeletal contractility in CAFs via the signaling molecules Yes-associated protein (YAP) and ROCK in a self-reinforcing positive feedback loop, by which CAFs maintain their differentiated phenotype [110]. Moreover, in vitro studies from our lab have shown that the stiffness of the ECM substrate influences not only the cytoskeletal αSMA-rich stress fibers but also the adhesion and proliferation of fibroblasts (Figure 2).

A stiff stroma and elevated Rho-dependent cytoskeletal tension promote focal adhesion formation, disruption of adherens junctions, and disturb tissue polarity [106,111,112]. In a striking study, Paszek et al. show that matrix stiffness is associated with integrin clustering, Extracellular signal–regulated kinase (ERK)-enhanced activation, and increased ROCK-generated contractility and formation of focal adhesions, in a mechanoregulatory circuit [106]. If this process becomes chronic, it promotes cell growth, disturbs tissue organization, and thus supports malignant transformation [106]. The desmoplastic response with enhanced matrix stiffening also influences the metastatic potential of epithelial cancer cells. Transformed cells often exert abnormally high forces, and these forces consequently disrupt cell–cell junctions, compromise tissue polarity, allow anchorage-independent survival, and ultimately increase invasion [49]. The cell-generated forces can also account for increased invadopodia, focal adhesion maturation and actomyosin contractility [49]. Tension-dependent matrix remodeling can also occur, as a consequence of increased contractility of tumor cells and CAFs, as it is observed in a reorientation of collagen fibrils surrounding the invasive front of the tumor [49]. Moreover, contraction of CAFs and tumor cells, and matrix stiffening cause high interstitial pressure which is another characteristic feature of the TME. Practically, the high tissue tension and high interstitial tension mechanically affects tumor vasculature by obliterating and provoking the collapse of blood and lymphatic vessels in the tumor [46,83].

Degradation of collagen and of other ECM molecules also contributes to tumor-induced ECM-remodeling and is another essential requirement for tumor invasion, where MMPs play a crucial role [113]. In mesenchymal cell migration, invading cells present focalized cell–matrix adhesions containing multi-molecular integrin clusters and increased proteolytic activity against ECM substrates [113]. Overexpression of MMPs-3, -11, -12, and -13 was detected in tumor stroma, along with MMP-2 in transformed mammary epithelial cells [49,114]. Furthermore, tumor cells recruit MMP-2- and MMP-9-producing neutrophils and macrophages [114]. Notably, immune cells tend to accumulate and migrate within dense collagen-enriched tumor stroma regions [115]. The activity of MMPs can be countered by both endogenous and pharmacological inhibitors. High expression of protease inhibitors (e.g., serpin family members) is associated with good prognosis, whilst tumors with high expression of integrins and MMPs correlate with poor prognosis and risk of recurrence [116]. Therapies employing pharmacological MMP inhibitors have been tested for various cancers with limited success so far [48].

Proteolytic fragmentation of ECM proteins not only leads to remodeling or degradation of the ECM scaffold, but also release defined ECM protein fragments, so-called matrikines, which act as soluble mediators such as cytokines and influence both cancer and resident cells of the tumor tissue. Moreover, they have attracted special attention as potential new anti-cancer agents [117]. Matrikines can block pathways that are involved in proliferation and invasion of tumor cells, and they affect angiogenic and lymphangiogenic processes [117]. Collagen XVIII-derived endostatin [118] and perlecan-cleaved endorepellin [119], strongly inhibit tumor growth in many preclinical cancer models and show angiogenesis-blocking effects on sprouting ECs [117].

## 4. Interactions of Cancer Cells with Endothelial Cells

### 4.1. Tumor Vascularization

In the prevascular phase of tumor dormancy, there is a dynamic equilibrium between proliferation and hypoxia-induced apoptosis of cancer cells [120]. The oxygen diffusion limit in tissue is around 150 μm which restricts avascular tumor growth to just a few millimeters [121]. When a tumor grows beyond this size, it flips an angiogenic switch and triggers an angiogenic cascade to recruit its own vasculature and connect to the blood circuit [122,123]. The vasculature becomes permanently activated to form new vessels by sprouting from pre-existing vessels in order to supply the tumor with blood and sustain its growth [9]. This angiogenesis is driven by numerous pro-angiogenic cytokines, chemokines, and matrix-degrading enzymes during tumor development [124,125,126,127]. In addition to tumor cells themselves, infiltrating bone marrow-derived monocytes that differentiate into tumor-associated macrophages (TAMs) [128] are a further source of angiogenic factors [129,130,131] that recruit endothelial and mural cells, such as pericytes [132,133]. From the microscopic premalignant phase onwards, this neovascularization enables the tumor to grow exponentially [9,122,134,135].

**Figure 3:**
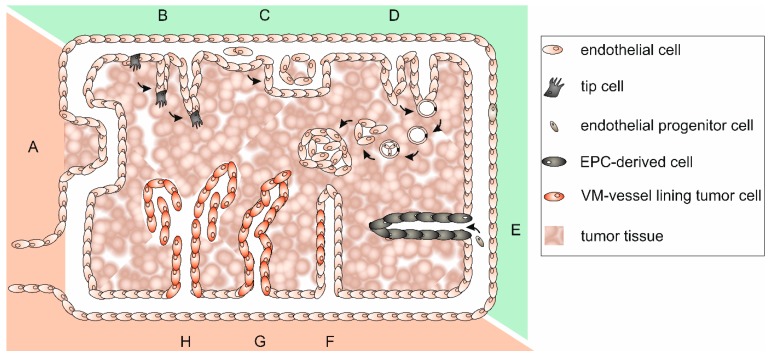
Different types of vascularization allow the blood supply of tumor tissue. Different types of vascularization can occur simultaneously and even merge: (**A**) co-option of preexisting vessels; (**B**) sprouting angiogenesis of endothelial cells; (**C**) intussusceptive angiogenesis; (**D**) glomeruloid angiogenesis; (**E**) vasculogenesis by recruitment of bone marrow-derived endothelial progenitor cells (EPCs); (**F**) in mosaic vessels, patches of tumor cells insert into the endothelium; (**G**) tubular type vasculogenic mimicry (VM) of tumor cells; and (**H**) patterned type VM of tumor cells. While angiogenesis (**B**–**D**), and vasculogenesis (**E**) depend on proliferation of ECs and bone marrow-derived EPCs, vessel co-option and VM (**F**–**H**) are EC proliferation-independent ways to support tumor growth. The recruitment of bone marrow-derived EPCs from distant parts of the body impairs radiation therapy, while vessel co-option and VM are unassailable to anti-angiogenic therapy. Vascularization mechanisms that are susceptible to anti-angiogenic therapy are highlighted in green, those that are insusceptible in red.

Tumor blood vessels appear little differentiated, highly tortuous, disorganized, and chaotic. This is why blood flow is disturbed and drug delivery hampered. Tumor vasculature is unexpectedly complex and can be classified into at least six types [136]. Its specific organization and the underlying tumor vascularization mechanisms have been reviewed in [127,137,138,139]. A lack of mural cells, a poorly formed BM and a discontinuous endothelium, in which even tumor cells may be incorporated, render the tumor vasculature leaky and also promote metastasis, The tumor vasculature-surrounding ECM is anomalously rich in the oncofetal fibronectin ED-B splice variant, which is synthesized by neoplastic cells [140,141], and in tenascin-C and -W, which are synthesized by melanoma and glioblastoma cells and by CAFs of most carcinomas [101,142]. Tenascin-C promotes the survival of tumor stem cells, inhibits immune surveillance, stimulates angiogenesis, proliferation, invasiveness, and metastasis of tumor cells [101,142]. Furthermore, Tenascin-C expressing neuroblastoma cells can transdifferentiate into tumor cell-derived ECs [143]. Tenascin-W is exclusively detectable in tumor stroma and can be used as a tumor marker for breast and colon cancer [102,144]. In addition to preexisting vessels that can be co-opted by tumor cells (Figure 3A), neovessel formation can originate from quiescent vasculature in various ways, which are collectively called tumor angiogenesis. This general term includes EC sprouting, intussusceptive and glomeruloid angiogenesis (Figure 3B–D). Vasculogenesis, in contrast, is a process of tumor neovascularization in which bone marrow-derived cells are recruited and differentiate into EPCs (Figure 3E). Thus, tumor ECs are heterogeneous and can originate from multiple sources [145]. Furthermore, cancer stem-like cells can accomplish vasculogenesis [146], and tumor cells themselves may differentiate to take over EC functions and line partly or even completely plasma containing conduits [147]. Integration of tumor cells into an EC layer forms mosaic vessels (Figure 3F), and the complete lining of blood-filled tubes with tumor cells is a process called vasculogenic mimicry (VM) (Figure 3G–H) [148,149]. These heterogeneous formation mechanisms together with the persistent tumor vessel growth lead to a constantly shape-changing, tortuous, and highly irregular tumor vasculature of which about 30% comprise arteriovenous shunts that bypass capillaries [120]. The consequential poor perfusion causes hypoxia of ECs, which hereupon release more pro-angiogenic molecules and stimulate further tumor angiogenesis [120]. The highly irregular architecture of the tumor vasculature together with irregular direction of flow, turbulences, and pressure conditions renders the tumor vasculature intrinsically leaky [150,151,152]. This causes an increased interstitial pressure, which makes it difficult for chemotherapeutics that are administered via the bloodstream to reach their site of action [153].

The proliferation of tumor cells alongside of preexisting vessels is termed vessel co-option and occurs predominantly early in tumor growth, although there is evidence that hijacking vessels by co-option might persist during all stages of tumor growth [137,154,155]. With progressive tumor growth, tumor cells proliferate around constantly formed neovessels which markedly differ from normal vessels in morphology and molecular composition [156,157]. Angiogenic sprouting of ECs, which are pivotal in blood vessel growth [158], is usually involved in the formation of these vessels [159]. Triggered by an angiogenic stimulus, select ECs differentiate into tip cells that migrate along a stimulatory gradient into the avascular ECM. Other ECs start to proliferate and form cord-like structures behind the tip cells. These cords develop into endothelial tubes that finally anastomose; pericytes and smooth muscle cells are recruited, and a new BM is formed [160,161,162]. New tumor vessels can also arise via EC columns that move into the vessel lumen, and these transluminal pillars enlarge and form new vessel walls that split the pre-existing vessel into two in a process called intussusceptive angiogenesis [163,164,165]. Neovascularization by intussusceptive rather than sprouting angiogenesis is energy-saving and faster, and occurs inter alia in gliosarcoma multiforme, melanoma, breast and colon cancer [166]. Glomeruloid angiogenesis, found in many aggressive tumors, is another way of tumor angiogenesis in which several microvessels are ensheathed by a BM of varying thickness containing few pericytes to form complex vascular structures termed glomeruloid bodies [137,167,168]. Additionally, there is evidence for vasculogenesis by recruitment of bone marrow-derived EPCs that differentiate into ECs [120,169,170,171]. EPCs also promote the angiogenic switch and the transition from micro- to macro-metastasis [172]. Furthermore, in many cancers highly invasive and genetically dysregulated tumor cells have been reported to adopt an EC-like phenotype [173,174] and form partially non-EC-lined mosaic vessels and even completely non-EC-lined vascular-like channels to support their own blood supply by VM [148,149]. Such VM channels can arise either by tubular or patterned matrix type VM [175,176]. While VM networks of the tubular type morphologically resemble the pattern of embryonic vascular networks [137,177], the morphology and topology of the patterned matrix type strongly differs from EC-lined vessels. It displays an intricate meshwork of extravascular patterned depositions of matrix proteins such as laminins, collagens IV and VI, and heparan sulfate proteoglycans that wrap around interdigitating and branching cylinders of tumor cells and, unlike fibrovascular septa, form hollows that anastomose with blood vessels [175,178,179]. All these types of vessel formation can occur in parallel, and also gradual transitions are possible. All of them comprise numerous sequential steps which crucially depend on integrins [127] and MMPs [31,180,181,182] as well as on soluble growth factors [183].

Once the tumor is connected to the vasculature, ECs become part of the tumor tissue and communicate with the other cells in the tumor tissue. Cancer progression is promoted when this communication goes awry [184,185]. At a later progression stage of a primary tumor, both angiogenetic and lymphangiogenic vessels allow tumor cells to disseminate and use the blood or lymph as a direct route of transportation to colonize distant organs. In this way, a cancer cell that successfully transmigrates through the endothelium into another tissue can form a metastasis. Regarding cancer invasion and metastasis, the endothelium acts rather as a launching site than as a barrier. ECs can affect the invasiveness of cancer cells by controlling their vascular dissemination [186] or by increasing their invasive capability [187].

Angiogenesis, vasculogenesis and vessel-based metastasis are controlled by cancer-endothelial cell (CEC) interactions. Different molecular modes of action underlying CEC interactions can be distinguished: (i) chemokine- and soluble factor-mediated interactions; (ii) tumor-endothelial communication via extracellular vesicles; and (iii) biomechanical (physical) interactions by, e.g., gap junctions and adheren junctions.

### 4.2. Soluble Factors Mediate CEC Interactions during Angiogenesis and Vasculogenesis

Tumor cell-secreted growth factors influence the TME and attract ECs. Such factors usually activate receptor kinases or ion channels to trigger an intracellular response. The most important endothelial growth and survival factors are the VEGFs. The VEGF family consists of five members (VEGF-A, -B, -C, and -D, and placental growth factor) that can bind to three tyrosine kinase receptors (VEGFR-1, -2, and -3) [188]. VEGF-A is the most significant inducer of local angiogenesis. Chronic VEGF stimulation in tumors promotes excessive sprouting and branching by tip cells leading to irregularities in the tumor endothelium and loss of its barrier function [189]. Almost all tumors express VEGF-A as essential growth factor in pathological angiogenesis. Furthermore, it is the prime elicitor of the angiogenic switch [190].

Originally identified as mediators of inflammatory diseases, chemokines link tumor and stromal cell communication networks to induce a proper microenvironment for tumor growth and metastasis [191]. Chemokines are a family of small cytokines secreted by cells. They bind to G protein-coupled chemokine receptors on target cells. CXCL12 is the most important CXC chemokine and is implicated in cancer cell extravasation and metastasis [192,193]. It is found in many tissues and in serum. Expressed by stromal cells of distant organs, CXCL12 promotes metastasis by attracting cancer cells and stimulating cancer cell extravasation, migration, and adhesion to ECM and to stromal cells. On cancer cells, it binds to and signals via CXC chemokine receptors type 4 (CXCR4) and 7 (CXCR7). A simultaneous and enhanced expression of CXCL12 and CXCR4 has been found in many cancers, such as breast [194], gastric [195], pancreatic [196,197], ovarian [198,199], cervical [200] and oral squamous cell carcinoma [191]. CXCL12 promotes the attachment of prostate cancer and breast cancer cells to ECs, and increases their transendothelial migration in vitro. Murakami et al. also demonstrated that ectopic expression of CXCR4 has similar effects on melanoma cells in vitro, and that it enhances lung metastasis in vivo [201].

Micro RNAs (miRNAs) are also significant regulators of angiogenesis and tumor metastasis. They are short (20–24 nucleotides) non-coding endogenous RNAs that occur in multicellular organisms and can influence the expression of many genes by post-transcriptional silencing or by causing the degradation of their mRNAs. miRNAs, which are frequently deregulated in many types of cancer, facilitate tumor growth, invasion, angiogenesis, and immune evasion through controlling translation of their target mRNAs [202,203]. For instance, in ECs co-cultured with hepatocellular carcinoma cells, three miRNAs, miR-146a, miR-181a*, and miR-140-5p, are upregulated, whereas miR-302c is downregulated [204]. Upregulation of miR-146a promotes EC migration and proliferation, as well as tumor growth and vascularization [204]. Furthermore, miRNAs can selectively be exported from cells in membrane-bound vesicles (exosomes and MPs), lipoproteins, and other ribonucleoprotein complexes. The content of these vesicles/particles varies with and corresponds to the (patho)physiological state distinct signature of the secreting cell. After the uptake of exosomal miRNAs by neighboring or distant cells, these miRNAs modulate the gene expression in the recipient cell [205]. Zhuang et al. have demonstrated that, via microvesicles, miR-9 transfers information from cancer to ECs. Thus, miR-9 supports angiogenesis and tumor growth [206].

### 4.3. Direct Tumor Cell–Endothelial Cell Interaction and Integration of Tumor Cells in Mosaic Vessels

Fifteen percent of vessels in xenografted and spontaneous human colon carcinomas have been reported to be of a mosaic type (Figure 3F) [207]. It is not yet clear whether these abnormal vessel structures are formed by cancer cells which integrate into the EC layer of the vessel wall or whether they arise by apoptosis of ECs and exposure of underlying cancer cells. Along with their incorporation into tumor blood vessels, cancer cells undergo epithelial–mesenchymal transition and acquire endothelial characteristics. The interaction between endothelial-like cancer cells (EndCC) and ECs, blood components, and inflammatory signals procures the differentiation of cancer cells into EndCCs. EndCCs interact with neighboring ECs, but they also possess migratory and invasive properties [208]. By biomechanical interaction of breast cancer cells with the endothelium, ECs stimulate proliferation, survival, and stemness of breast cancer cells and thus metastatic dissemination [209].

Gap junctions are special channels through the plasma membrane that directly connect the cytoplasms of neighboring cells and thus mediate short-range and direct intercellular communication which is necessary for proper tissue development and homeostasis [210]. They consist of transmembrane proteins of the connexin family [210] and allow free diffusion of small molecules and ions, and also the transport of miRNAs and small interfering RNA (siRNA) silencing signals [211,212] between cells. Altered expression of gap junction proteins is an important step in carcinogenesis [213]. Moreover, connexins play a crucial role in the direct cellular communication between cancer cells and ECs [214,215,216,217]. Extravasating breast cancer cells induce in ECs tyrosine phosphorylation of connexin 43 which facilitates further tumor cell extravasation [218]. The gap junction inhibitor, oleamide, significantly decreases homotypic communication between cancer cells and also heterotypic interaction between cancer cells and-ECs. Oleamide treatment in vitro attenuates the expression levels of several angiogenic factors, such as VEGF, HIF-1α, CXCR4, Cx26, Cx43, and MMP-9, presumably via an impaired connexin-mediated intercellular communication [219].

ECs are tightly connected via VE-cadherin-containing adherens junctions [220,221,222]. VE-cadherin’s C-terminus is linked via β-catenin or plakoglobin to the actin cytoskeleton [223]. Blocking VE-cadherin by monoclonal antibodies inhibits angiogenesis, tumor growth, and metastasis [224]. Endothelial barrier integrity depends on differential phosphorylation of six out of nine tyrosine residues in the cytoplasmic tail of VE-cadherin [225,226]. Especially phosphorylation of Y658 and Y731 decreases vessel tightness [227]. Different cancer types vary with respect to VE-cadherin phosphorylation in neighboring ECs, which differentially affects cancer metastasis [228,229,230].

## 5. Tumor Cells Imitating Endothelial Cells in Vasculogenic Mimicry Vessels

### Vasculogenic Mimicry and Its Molecular Phenotypes

**Figure 4:**
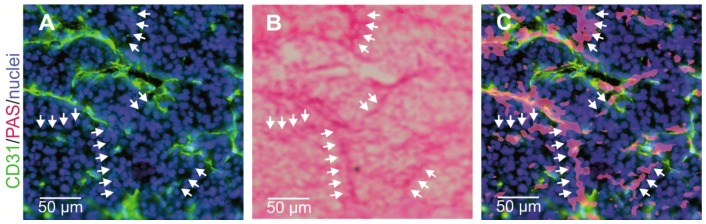
Vasculogenic mimicry of cancer cells lining tumor vessels. CD31-negative/PAS-positive VM channels in a HT1080 xenograft mouse tumor model were visualized by consecutive immunostaining and histochemical staining of the same cryosection: (**A**,**C**) normal CD31-positive blood vessels are labeled in green; and (**B**,**C**) CD31-negative VM channels are detectable by PAS staining. Nuclei are stained blue. (**A**) Cryosections were first immunostained and photographed; (**B**) subsequently, histochemically PAS-stained and photographed again; and (**C**) then the images were overlaid to demonstrate numerous CD31-negative/periodic acid Schiff (PAS)-positive VM channels (arrows). Representative images are shown.

Vasculogenic mimicry as one form of neovascularization was first described by Maniotis et al. [148]. Unlike angiogenesis and vasculogenesis, VM does not depend on ECs, but tumor cells themselves form vascular channels to support at least the supply with oxygen and nutrients. Since the first report of VM in 1999, its existence was controversially debated [231]. Notwithstanding, VM is clearly associated with tumor aggressiveness, and poor prognosis [232,233]. VM channels are typically characterized as an intricate meshwork of micro-channels of irregular diameter that anastomose with endothelium-lined blood vessels, but in contrast to them they are devoid of endothelial markers such as CD31. Simultaneously, they are covered by extravascular depositions of glycosylated matrix proteins, such as laminins, collagens IV and VI, and heparan sulfate proteoglycans that are positive for periodic acid Schiff (PAS) staining (Figure 4) [175,178,179,234]. Continuity and anastomosis with endothelium-lined normal vessels is a prerequisite for the functional significance of such VM channels [231], together with red blood cells in their lumen [148]. Moreover, it is conceivable that VM channels, which are too small to transport red blood cells, could also supply tumor tissue with nutrients and oxygen by hemoglobin from ruptured erythrocytes [147,235]. In a murine xenograft tumor model of inflammatory breast cancer, tumor cell lines that either do or do not show VM were used. Thus, VM channels could be discriminated from other tumor vasculature by three-dimensional contrast-enhanced dynamic micro-Magnetic resonance imaging (MRI) with G6-(1B4M-Gd)_256_ dendrimer as contrast agent [236,237]. Meanwhile, VM has been observed in more than fifteen cancers, such as astrocytoma World Health Organization (WHO) grade II–III [238], glioblastoma (astrocytoma WHO grade IV) [177], melanoma [239,240], cancers of breast [237], gallbladder [241], pancreas [242], liver [243], esophageal [244], gastrointestinal [245], and colorectal tract [246], lung [247,248], ovaries [249,250], prostate [251], and various sarcomas [252,253]. In multiple myeloma, bone marrow macrophages and mast cells are additionally involved in VM of bone marrow vascularization [254,255].

Unlike the prediction based on numerous preclinical models, tumors are very likely to acquire an intrinsic resistance to angiostatic drugs [256,257]. Moreover, extrinsic mechanisms can contribute to resistance, demonstrating the important role that stromal cells play in the context of tumor neovascularization [258]. Tumor cells release many chemokines, inter alia the pro-angiogenic factors CCL2, CCL5, and CXCL12 [259] and cytokines, among them redundant pro-angiogenic cytokines, such as basic fibroblast growth factor (bFGF), interleukin-8 (IL-8), hepatocyte growth factor (HGF), PDGF, and VEGF [260], which are difficult to inhibit simultaneously. Furthermore, a tumor’s blood supply by non-angiogenically originated vessels (Figure 3) is also not impaired by anti–angiogenic treatment [261]. The tumor stroma contains many different cells, among them ECs, mural cells, platelets, CAFs, and TAMs, whose roles in resistance to angiostatic therapy have been reviewed recently [258]. Similar to mesenchymal stem cells, which are capable of tubulogenesis in vitro [262], it appears that some aggressively growing tumor cells can phenotypically mimic or transdifferentiate into several of these cell types, e.g., they can adopt features of ECs [173,174], pericytes [263,264], and even platelets [265,266,267]. In initiation of VM, both EMT and tumor-initiating cancer stem-like cells (CSCs) play important roles [149,268,269]. In glioblastoma, a portion of the tumor vasculature arises from CSCs which have been reported to differentiate to tumor vessel pericytes upon CXCL12/CXCR4 and TGFβ signaling [263]. Macrophage migration inhibitory factor (MIF) also triggers via CXCR4 and AKT EMT in glioblastoma [270]. However, there are conflicting data whether CSCs transdifferentiate into ECs and/or pericytes [271].

To produce a functional tumor vasculature, many signaling molecules and pathways interact in a complex network, and the molecular regulation of tumor angiogenesis has been reviewed earlier to indicate therapeutic possibilities [272,273,274]. VM exhibits multiple molecular phenotypes, because several signaling pathways are interconnected here which are involved in vascular and embryonic/stem cell differentiation and in adaption to hypoxic conditions [174,275,276,277]. In adaption to the hypoxic conditions prevailing in tumor tissue, HIFs are crucially responsible. The HIF-driven pathways have been recently reviewed [278,279]. Hypoxia-response elements (HREs) are involved in regulating cell proliferation, cell death, angiogenesis, blood vessel co-option, cell adhesion molecules, secretion of MMPs, antigen presentation mechanisms and immunosuppressive factors, and additionally in vasculogenic mimicry [280]. Under normoxic conditions the α-subunit (HIF-1α, HIF-2α or HIF-3α) of the hypoxia-induced transcription factor HIF is rapidly degraded in the cytosol, whereas under hypoxic conditions it binds to the constitutively present β-subunit, thus forming an active heterodimer that translocates to the nucleus, where it controls gene expression by binding to HREs ([279] and references therein). While the transcription factor HIF-1α plays an important role in promoting sprouting angiogenesis [281], HIF-2α promotes EMT and thus VM in pancreatic cancer [282]. This is in line with the observation that VM is especially found in a hypoxic tumor core [283]. In a neuroblastoma model, an immunotherapy targeting tumor-derived ECs failed, because the treatment increased hypoxia, causing further EMT and tumor-derived EC trans-differentiation, and adaptation to the hypoxic microenvironment [284].

Hypoxia modulates the expression of many genes involved not only in angiogenesis, but also in VM, inter alia VEGF-A, VEGFR-1, Erythropoietin-producing human hepatocellular (EPH) receptor A2 (EphA2), TWIST, COX-2, and Nodal [285]. The transcription factor HIF-2α promotes EMT in pancreatic cancer by upregulating the transcription factors TWIST1 and TWIST2 in carcinoma cells which then upregulate VE-cadherin [282] and downregulate E-cadherin respectively [286]. Such VE-cadherin-expressing carcinoma cells may readily incorporate into the endothelium and give rise to composite vessels and eventually VM. Furthermore, under the selection pressure imposed by hypoxia, polyploid giant colorectal cancer cells have been reported to express EMT-related genes, to become pluripotent, and to give rise to erythroid cells expressing embryonic and fetal hemoglobin, and also to acquire EC-like features to form VM channels [287,288]. Furthermore, peroxiredoxin 2 (PRDX2), a major antioxidant enzyme, stimulates VM channel formation in colorectal cancer by keeping VEGFR-2 in its activated state [289]. The VM phenotype is thus associated with transdifferentiation of CSCs and cell plasticity [276,290], and VM channel-lining tumor cells phenotypically mimic ECs. However, they differ from ECs regarding their expression of TIE-1, VEGF-C, neuropilin.1 (NRP1), endoglin, Tissue factor pathway inhibitor (TFPI1), Laminin subunit γ2 (LAMC2), and EphA2, whereas they do not express Tyrosine kinase with immunoglobulin-like and EGF-like domains 2 (TIE-2), VEGFR-1, VEGFR-2, P-selectin, vascular adhesion protein-1 (VCAM-1), and CD31 [276].

Important transcription factors for the expression of VM-relevant genes are TWIST1 and BMI1, which are also relevant for EMT [291,292]. The EMT marker TWIST1 is activated by B-cell lymphoma 2 (Bcl-2) [293] and by metadherin (MTDH) [294], which drives CSC expansion and VM. Furthermore, CCL21/CXCR7 signaling activates the transcription factor SNAI2/Slug via ERK and Phosphatidylinositol-4,5-bisphosphate 3-kinase (PI3K)/AKT signaling in chondrosarcoma, and thus promotes EMT [295]. Together with TWIST1 and the Snail family transcription factors SNAI1/Snail and SNAI2/Slug, the Zinc finger E-box-binding homeobox 1 proteins ZEB1 and ZEB2 are pivotal EMT regulators with significant overlap in their signaling networks [296]. ZEB2, triggered by TGFβ1, promotes cell motility, invasiveness, expression of EC markers, and formation of VM vessels in hepatocellular carcinoma [296]. The paired-related homeobox transcription factor 1 (Prrx1) is also implicated in EMT, but although it is co-expressed and cooperates with TWIST1 in EMT, it suppresses stemness properties of cancer cells, and thus uncouples EMT and stemness [297]. In VM channel formation and differentiation, VE-cadherin [276], erythropoietin-producing hepatocellular receptor A2 (EphA2) [298], phosphatidyl inositol 3-kinase (PI3K) [298], MMPs [299], VEGFR-1, and HIF-1α are instrumental [174,300]. The migration inducting gene Mig-7 is expressed early in placenta development during maximal cytotrophoblast invasion and vascular remodeling, and also by carcinoma cells, where it is linked to VM [301,302]. Focal adhesion kinase (FAK) and Mig-7 induce upregulation of MMP-2 and MMP-9, which are involved in ECM degradation and VM [301,303,304,305].

The laminin binding lectin galectin-1 [306] is overexpressed on tumor-associated ECs and in their surrounding ECM [307]. It is also involved in the interaction of regulatory T (Treg) cells with dendritic or T cells, and it is upregulated in Treg cells upon T cell receptor activation [308]. In squamous cell carcinoma ECs, galectin-1 is overexpressed and binds directly to neuropilin-1 (NRP1), thereby enhancing phosphorylation of VEGF-R2 and triggering signaling via Mitogen-activated protein (MAP) kinases SAPK1/c-Jun N-terminal kinase (Jnk), which increases EC proliferation and adhesion, and in combination with VEGF-A it enhances cell migration [307]. The likewise laminin-binding galectin-3 [309] essentially promotes VM in melanoma by upregulating in melanoma cells the ectopic expression of genes that are otherwise typical for ECs, such as VE-cadherin, IL-8, fibronectin-1, endothelial differentiation sphingolipid G-protein receptor-1 (EDG-1), and MMP-2 [310]. While MMP-2 creates fragments from laminin-332 that increase EGFR and F-actin expression and promote VM in large cell lung cancer, MMP-13 counteracts VM by releasing different laminin-332 fragments that decrease expression of EGFR and F-actin [311]. Increased NRP1 expression upon upregulation of VEGFA, secretion of MMP-2 and -9, and activation of αvβ5 integrin furthermore correlates with tumor cell invasiveness and VM [312,313]. Elevated NRP-1 expression levels are also implicated in development of resistance to anti–angiogenic therapy with VEGF-A blocking antibodies [314]. This may be due to the fact that NRP1 is not only a coreceptor of VEGFR-2 for VEGF-A but also signals upon binding of other growth factors such as class 3 semaphorins, TGFβ, HGF, FGF, and PDGF [315]. Upon PDGF-C stimulation, NRP-1 triggers invasion and VM of VEGFR-and PDGFR-deficient melanoma cells [313].

Nodal plays an essential role in VM such as in embryonic/stem cell differentiation as demonstrated by an impaired VM of aggressive melanoma cells upon downregulation of Nodal [174,316]. Notch 1 triggers EMT in hepatocellular carcinoma and promotes VM [317], while Notch4 is highly expressed in melanoma CSCs, where it promotes metastasis via the TWIST/VE-cadherin/E-cadherin pathway [269].

In addition to transcription factors, miRNAs are involved in post-transcriptional regulation of VM, thereby modulating tumor angiogenesis and cancer metastasis. TWIST1 upregulates 18 miRNAs in hepatocellular carcinoma cells, among them miR-27a-3p which targets VE-cadherin and suppresses EMT and VM [318]. Pointing in the same direction, miR-27a negatively regulates the expression of EphA2, SNAI1, and SNAi2 [319]. miR-27b binds to the 3′-untranslated region (3′-UTR) of VE-cadherin mRNA and inhibits ovarian cancer cell-mediated VM through suppression of VE-cadherin expression [320]. Loss of miR-26b promotes VM by increased EphA2 expression in glioma [321]. miR-124 regulates the expression of several EMT- and VM-relevant genes, such as CD151, ROCK1, integrin β1, Rac1, SNAI2, and angiomotin-like protein 1 (AMOTL1) [322,323,324,325,326]. TWIST1 downregulates miR-26b-5p in hepatocellular carcinoma by binding to its promotor region, thereby unchecking Smad1 expression and deregulating BMP4/Smad1 signaling, which promotes EMT [327]. miR-26-5p in hepatocellular carcinoma is a negative regulator of VE-cadherin, SNAI1, and MMP-2, and thus VM [328]. miR-186 downregulates the expression of TWIST1 in prostate cancer and thereby among other effects inhibits EMT and VM [329]. Loss of miR-4638-5p promotes VM in castration resistant prostate cancer by activating PI3K/AKT signaling via the kinase D-interacting substrate of 220 kDa (KIDINS220) scaffold protein [330]. KDKDM4b hypermethylates the miRNA-615-5p promotor in hepatocellular carcinoma, thereby epigenetically silencing this miRNA and consecutively increasing expression of the Ras-related protein RAB24, which activates the Rab-Ras-pathway and promotes adhesion, EMT, and VM [331].

Long non-coding RNAs (lncRNAs) are a recently discovered class of gene regulators in many physiological and pathological processes [332], and by their interaction with miRNAs [333] they are involved in metabolic reprogramming and EMT [334,335]. lncRNAs and their interaction with miRNAs in EMT have been reviewed recently [333]. The oncogenic lncRNA metastasis-associated lung adenocarcinoma transcript 1 (MALAT1) is implicated in tumor angiogenesis and also in VM by upregulating the expression of VE-cadherin, β-catenin, MMP-2, MMP-9, MMP-14, p-ERK, p-FAK, and p-paxillin [336], by upregulating N-cadherin and fibronectin, and by suppressing E-cadherin [113].

To maintain an anti-coagulatory milieu in VM vessels, channel lining tumor cells can upregulate the expression of tissue factor (TF), TF pathway inhibitor-1 (TFPI-1), and TFPI-2 [337]. VM channels not only supply the tumor with oxygen and nutrients but also might, to a limited extent, aid in some of the draining function of lymphatics [179,337,338].

Tumor growth and metastasis are promoted by angiogenic and vasculogenic pathways as well as by vessel co-option and VM. The latter two are notorious for conveying drug resistance. VM occurs in many, albeit not all, tumor tissues, but not in the healthy body, although some authors believe that hypoxic trophoblasts in placenta tissue are able to contribute to their own blood supply by VM [279]. VM correlates with a poor prognosis [247,339], because it promotes cancer growth and hematogenic dissemination of detaching tumor cells leading to metastasis [300,340,341,342,343,344]. In colorectal cancer, VM is positively associated with invasion depth, lymph node metastasis, distant metastasis and tumor-node-metastasis stages and negatively with patients’ overall survival [345]. Likewise in ovarian carcinoma, VM is associated with tumor and lymph node metastasis grade, implantation, and stage, and with reduced patients’ overall survival [346]. In ovarian carcinoma, VM correlates with the immunohistochemical detection of ALDH1, Kisspeptin (KiSS-1), and Metastasis associated in colon cancer-1 (MACC1), which are used to predict metastasis and prognosis, and VM proved to be a prognostic marker, as well as a potential target to treat epithelial ovarian carcinoma [346]. Similar data have been reported for colorectal carcinoma [345]. In addition, in non-small cell lung cancer, VM, promoted by Dickkopf-related protein 1 (DKK1) is associated with poor differentiation, advanced stage, and distant metastasis [347]. In hepatocellular carcinoma, both tubular and patterned type VM have been reported, and the latter has been ranked as an unfavorable prognostic marker [348].

## 6. Perspective: New Cancer Therapies Targeting Tumor Vasculature and CAFs

### 6.1. Anti-Angiogenesis and Normalization of the Tumor Vasculature

Cancer therapy comprises surgery, radio- and chemotherapy, targeted therapy, immunotherapy, hypothermia, hormone therapy, stem cell therapy and combinations of these methods [349]. Radiation therapy and chemotherapy target both cancer cells and tumor vasculature. Bone marrow-derived cells can restore radiation-damaged blood vessels, and they can support surviving tumor cells [272]. In addition, EPCs between the smooth muscle and adventitial layer of vessel walls, may trigger tumor neo-vascularization [156,272].

Endostatin and other anti–angiogenic inhibitors specifically target ECs rather than tumor cells to inhibit tumor angiogenesis. Such an anti–angiogenic therapy has four advantages over the usually applied cytotoxic chemotherapeutic drugs [272]: (i) angiogenesis is a homogeneous process, and therefore its inhibition should be effective in any solid tumor; (ii) an anti–angiogenic therapy approach is not impaired by tumor cells that become resistant to chemo- or radiation therapy; (iii) ECs can be directly targeted with blood-borne drugs without the need to counteract the usually high tumor interstitial pressure; and (iv) the tumor vasculature can be specifically targeted due to a differentially upregulated expression of receptors on tumor ECs versus normal EC. Therefore, anti–angiogenic therapies targeting VEGF family members, their receptors, or other pro-angiogenic factors raised high expectations [350]. However, they have not yet produced the clinical benefits initially envisioned [351].

In contrast to anti–angiogenesis, “vascular normalization” returns malformed and dysfunctional tumor vessels into vessels with a similar appearance and functionality as in normal tissues. It aims to overcome the serious problems arising from: (i) the physical barrier of tumor vessel walls; (ii) the high interstitial pressure in tumors; and (iii) the acquisition of drug resistance by genetic or epigenetic mechanisms [153]. However, delivery of chemotherapeutics may be impeded by an impervious endothelial layer [352]. Tumor-vascular disruptive agents induce a tumor-selective breakdown of the vessel wall barrier, and a combined targeting of both tumor vasculature and tumor cells may increase the efficacy of chemotherapeutics [250,353]. Until now, strategies to normalize tumor vasculature did not yet meet the initially high expectations [354], and other strategies are sought.

When anti-VEGF-induced vascular normalization ceases to be effective, the tumor becomes resistant to additional anti–angiogenic therapy and grows even more aggressive, for not yet understood mechanisms [355,356]. Various reasons may underlie this resistance to anti–angiogenic therapy and may occur simultaneously: Anti–angiogenic treatment-induced hypoxia may increase the production of other redundant angiogenic factors or the invasiveness of tumor cells. Tumor cells may also acquire mutations that render them tolerant to hypoxia. Moreover, some anti–angiogenic therapies lack specificity and have toxic side effects [273,274,357]. Such a development of drug resistance after initial success and even more aggressive tumor growth was not anticipated [358,359], and anti–angiogenic treatment turned out to have promised too much for various reasons [134,360,361,362]. Even if angiogenesis is curbed, neovascularization of tumor tissue may occur by other modes such as intussusceptive or glomeruloid angiogenesis, by CSC-promoted vasculogenesis, or even by VM of tumor cells [363]. In addition, vessel co-option confers resistance to anti–angiogenic therapy [155]. Moreover, many other tumor stromal cells, such as ECs, mural cells, platelets, CAFs, and TAMs, can contribute to the development of resistance to angiostatic therapy [258]. The balance between ECs on the one hand side and stromal fibroblasts and inflammatory cells, which release many cytokines and angiogenic factors other than VEGF, on the other hand could be disturbed by anti-VEGF therapy [123,261,364]. In this sense, CAFs even have been denounced Trojan horse-like mediators of resistance to anti-VEGF therapy [365].

### 6.2. VM Channels Are a Promising New Therapeutic Target

The so far little considered concept of VM as a new therapeutic target structure attracts increasing interest [276], because in VM channels tumor cells line the vasculature and hence are directly amenable to therapeutics from the bloodstream [276,366,367]. They have been suggested as targets for vascular disrupting agents, drug delivery, and antitumor therapy [136,353,368]. A combination of either VM inhibitors or VM disruptive agents with anti–angiogenic therapies may be promising, even if targeting VM channels, that show great diversity with respect to cellular phenotype in diverse tumors, is not as universally applicable as EC-targeting therapies, that aim at largely uniform ECs [300].

**Figure 5:**
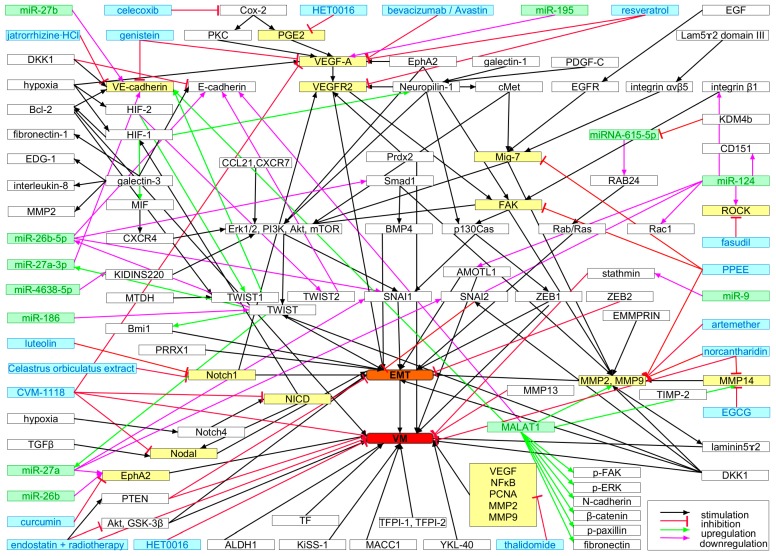
Molecular phenotype-defining signaling pathways in vasculogenic mimicry (VM). Signaling molecules that have been targeted to inhibit VM are highlighted in yellow and targeting compounds are marked in blue. Regulatory miRNAs are labeled green. EMT (highlighted in orange), which is pivotal for VM, and VM (highlighted in red) are the focal points in which all these signaling pathways converge. For details and references, see text.

By now, numerous VM-characteristic molecular determinants and signaling pathways have already been delineated [276,278,366,369]. Tumor cells isolated from malignant pleural effusions, which develop in various malignancies due to impaired fluid drainage by blood or lymphatic vessels, inflammation and increased vascular permeability and are routinely drained for diagnosis, have been employed to test VM tube formation in vitro. Such cells may help to pinpoint drugable molecular targets (Figure 5) and to develop and optimize personalized therapy [370]. In addition, a standardized assay, which in vitro recreates the formation of fluid conducting VM channels by cancer cells surrounding a glycoprotein-rich inner layer, may be instrumental in finding and characterizing VM targeting drugs [371]. Potential molecular targets of special interest may be EMT-inducing transcription factors (EMT-TFs), such as TWIST1, SNAI1/2, and ZEB1/2 (Figure 5) [296], where ZEB2 is not only an EMT regulator but also involved in VM [372]. Expression of the transcription factor high mobility group box-1 (HMGB-1), that also interacts with nucleosomes and histones [373], is upregulated by anti–angiogenic treatment [284]. Hence, HMGB-1 has been proposed as a target for tumor therapy [374].

In addition, migration-inducting gene 7 (Mig-7), which is involved in VM by carcinoma cells, but not expressed in normal cells, may be a promising target in VM channels [302], and Mig-7-inhibitory agents together with anti–angiogenic or other conventional anti-cancer drugs might act synergistically [301,302]. The extracellular matrix metalloproteinase inducer (EMMPRIN, CD147) may also be a potential target for anti–angiogenic therapy in glioma [375]. The angiogenic factor YKL-40 (human cartilage glycoprotein HC-gp39, CHI3L1) is produced by cancer cells, inflammatory cells, and stem cells [376]. By transdifferentiation of glioma stem-like cells into vascular pericytes/smooth muscle cell- and EC-like cells, YKL-40 promotes both angiogenesis and VM [377], which in a xenograft tumor model is susceptible to treatment with a neutralizing monoclonal antibody against YKL-40 in combination with radiation therapy [378].

More than ECs, cancer cells may be responsible for drug resistance to anti–angiogenic therapy [174]. Especially in VM developing tumors, VM channels lacking ECs are at least partially responsible for resistance to VEGF inhibition [379] or to anti-angiogenic agents, inter alia angiostatin and endostatin [367]. However, endostatin combined with radiotherapy suppresses VM formation through inhibition of EMT in esophageal cancer [380]. HET0016 (*N*-Hydroxy-*N*′-(4-butyl-2-methylphenyl)-formamidine), which was initially characterized as a selective inhibitor of 20-HETE (20-hydroxy-5,8,11,14-eicosatetraenoic acid) formation from arachidonic acid [381], can be used to target VM channels, whose formation is triggered by the small molecule proteinase kinase inhibitor vatalanib, which is used as anti–angiogenic therapeutic, [283]. Norcantharidin (3,6-endoxohexahydrophthalic anhydride), a demethylated derivative of cantharidin [382] downregulates MMP-9 via NFκB in hepatocellular carcinoma cells in vitro [383,384]. In vivo it also downregulates MMP-2 in a human melanoma mouse model [385], and MMPs-2 and -14 in gallbladder cancer, thereby enhancing the VM-inhibiting activity of TIMP-2 [386]. Mosaic vessel and VM channel formation in a B16F10 mouse melanoma model are reduced by thalidomide which inhibits expression of VEGF, NFκB, PCNA, MMP-2 and MMP-9 [387]. In addition, natural products with anti–angiogenic and anti-VM activity are very important for the development of new drugs. Such natural compounds and their molecular modes of action have been reviewed recently [388]. Genistein inhibits the expression of VEGF-A, PDGF, TF, urokinase-type plasminogen activator (uPA), and MMPs-2 and -9, whereas it stimulates expression of PAI-1, endostatin and angiostatin, as well as thrombospondin-1 [389]. In vivo, compounds such as genistein, jatrorrhizine hydrochloride, and curcumin inhibit VM in uveal and choroidal melanoma, respectively, via regulating VE-cadherin and EphA2 expression [390,391,392], whereas the antioxidant resveratrol has been reported to suppress VM in a murine melanoma model by decreasing the expression of VEGF and its receptors 1 and 2 [393]. The latter observation is in line with the finding that luteolin, likewise an antioxidant, inhibits Notch1-VEGF signaling and thus reduces VM formation in gastric cancer cells [394]. In addition, in a human hepatocellular carcinoma mouse model using GFP-labeled MHCC97-H cells, an ethnopharmacologically used *Celastrus orbiculatus* extract containing 11 terpenes, of which the effective component is not yet known, reduces VM formation by targeting Notch1 signaling [395]. An also not yet fully characterized ethanolic extract from *Paris polyphylla* has been reported to inhibit VM in a human osteosarcoma mouse model by downregulating the expression of FAK, Mig-7, and MMPs-2 and -9 [396]. Furthermore, inhibition of MMP-14 and tumor angiogenesis in two murine sarcoma and colon carcinoma models has been reported for the green tea ingredient (−)-epigallocatechin gallate (EGCG) [397].

Tumor vasculature targeting drug delivery systems have been reviewed recently, inter alia VM targeted approaches [398]. Targeting liposomes to endocytosis-prone surface receptors with ligand derivatives or antibodies improves the cellular internalization of encapsulated drugs. In combination therapy, liposomes and especially passive and active ligand-targeted liposomes have turned out to be efficient co-delivery systems for hydrophilic and lipophilic chemotherapeutic agents, such as drugs, anti-cancer metals, and gene agents [349]. Liposomes functionalized with a mannose-vitamin E derivative conjugate and a dequalinium lipid derivative to cross the blood brain barrier (BBB) and loaded with both the antimalarial drug artemether, as a regulator of apoptosis and VM channels, and the anticancer drug paclitaxel have been demonstrated in brain glioma-bearing rats to eliminate CSCs and tumor cells, and also to destroy VM channels [399]. In addition, aptamer-conjugated peptides allow delivering chemical drugs and gene drugs, e.g., antagomirs, simultaneously, as was demonstrated by co-delivery of the VM blocking ROCK inhibitor fasudil and VEGF inhibiting miR-195 [400].

### 6.3. Therapeutic Potential of Targeting CAFs

As CAFs are such central players in the tumor stroma, understanding the effect of CAFs on therapy and the development of a CAF-directed remedial treatment are of utmost importance as well. Indeed, CAFs affect irradiation therapy, as damaged or irradiated CAFs support tumor cell growth stronger than non-treated CAFs, possibly through up-regulation of cMet expression or its phosphorylation and MAP kinase activity in cancer cells [401]. Moreover, tumor stromal CAFs contribute to an increased intratumoral interstitial pressure, due to their potential to contract and to exert force on the ECM, thus compressing the interstitial space. This eventually results in attenuating therapeutic efficiency [46]. The interaction between cancer cells and CAFs can also reduce cytotoxic effects of chemotherapeutic drugs such as cisplatin by cell–cell adhesion through N-cadherin that activates the survival-promoting protein kinase B (PKB)/AKT and blocks pro-apoptotic Bad [402]. However, a clinical trial in which the Hedgehog signaling pathway was targeted and the tumor-induced mesenchyme activation was affected, did not show any therapeutic benefit [48].

## 7. Conclusions

As invasive cancer rates worldwide are continually increasing due to increased life expectancy, changes in lifestyle and nutrition, and environmental factors, cancer treatment is of prime importance. VM, albeit usually viewed as a negative prognostic marker, may constitute a potential new target for anti–angiogenic therapy [261,363]. VM and CAFs are not only passive bystanders but also active players within the tumor stroma, which contribute to tumor progression and dissemination. A better understanding of their molecular phenotypes and of their supportive roles for cancer cells are indispensable for pharmacological intervention, to resolve the burning issues of resistance to chemotherapeutic drugs and anti–angiogenic therapies, and to develop multimodal anti-angiogenic, anti-VM, and anti-proliferative strategies [138]. While tumors frequently develop resistance to anti–angiogenic drugs, new strategies that combine an anti–angiogenic therapy with a VM- or CAF-targeting approach may improve treatment success.

## Abbreviations

Akt	Protein kinase B
AMOTL1	Angiomotin-like protein 1
ARF	ADP ribosylation factor
αSMA	α-Smooth muscle actin
Bcl-2	B-cell lymphoma 2
bFGF	Basic fibroblast growth factor
BM	Basement membrane
Bmi1	B lymphoma Mo-MLV insertion region 1 homolog
BMP	Bone morphogenetic protein
CAF	Cancer-associated fibroblast
CD	Cluster of differentiation
CEC	Cancer-endothelial cell interaction
CHI3L1	Chitinase-3-like protein 1
CSC	Cancer stem-like cell
cMET	Hepatocyte growth factor receptor
c-Myc	Cellular Myelocytomatose (transcription factor)
COX-2	Cyclooxygenase-2
CXC	Cysteine-any amino acid-cyteine motif
CXCL12	C-X-C motif chemokine 12 = stromal cell-derived factor 1 (SDF-1)
CXCR4	C-X-C chemokine receptor type 4
DKK1	Dickkopf-related protein 1
EC	Endothelial cell
ECM	Extracellular matrix
EDA	Extra-domain A fibronectin splice variant
EDB	Extra-domain B fibronectin splice variant
EDG-1	Endothelial differentiation sphingolipid G-protein receptor-1
EGCG	(−)-Epigallocatechin gallate
EGF(R)	Epidermal growth factor (receptor)
EMMPRIN	Extracellular matrix metalloproteinase inducer
EMT	Epithelial–mesenchymal transition
EndCC	Endothelial like cancer cell
EPC	Endothelial progenitor cell
EphA2	Erythropoietin-producing human hepatocellular (EPH) receptor A2
Erk	Extracellular signal–regulated kinase
FAK	Focal adhesion kinase
FGF(R)	Fibroblast growth factor (receptor)
GAPDH	Glyceraldehyde 3-phosphate dehydrogenase
Glc	Glucose
Gln	Glutamine
GLUT2	Glucose transporter type 2
GSH	Glutathione
HGF(R)	Hepatocyte growth factor (receptor), cMet
HIF	Hypoxia-inducible factor
HRE	Hypoxia-response element
IL	Interleukin
Jnk	c-Jun N-terminal kinase
KDM4b	Lysine-specific demethylase 4B
KIDINS220	Kinase D-interacting substrate of 220 kDa
KiSS-1	Kisspeptin
LAMC2	Laminin subunit γ2
Lam5g2	Laminin-332 γ2chain
LOX	lysyl oxidase
MACC1	Metastasis associated in colon cancer-1
MALAT1	Metastasis-associated lung adenocarcinoma transcript 1
MCP1	Monocyte chemotactic protein
Mig-7	Migration-inducing gene 7
miR	Micro RNA
MMP	Matrix metalloproteinase
MP	Microparticle
MRI	Magnetic resonance imaging
MTDH	Metadherin
NADPH + H^+^	Nicotinamide adenine dinucleotide phosphate
NFκB	Nuclear factor κ-light-chain-enhancer of activated B cells
NICD	Notch intracellular domain
NRP1	Neuropilin-1
p130Cas	Cellular apoptosis susceptibility protein of 130 kDa
PAS	Periodic acid Schiff
PDGF	Platelet-derived growth factor
PEP	Phosphoenolpyruvate
PI3K	Phosphatidylinositol-4,5-bisphosphate 3-kinase
PK-M2	pyruvate kinase isoform M2
PPEE	*Paris polyphylla* ethanol extract
Prdx2	Peroxiredoxin-2
PRRX1	Paired-related homeobox transcription factor 1
ROCK	Rho-associated protein kinase
Rab	Ras superfamily of monomeric G protein
Rac1	Ras-related C3 botulinum toxin substrate
RANKL	Receptor activator of nuclear factor κ-B ligand
Ras	Rat sarcoma protein
ROCK	Rho-associated protein kinase
ROS	Reactive oxygen species
Smad	Small body size/mothers against decapentaplegic protein
SNAI	snail family transcriptional repressor
TAM	Tumor-associated macrophage
TCA	Tricarboxylic acid
TF	Tissue factor
TFPI1	Tissue factor pathway inhibitor
TGFβ1	Transforming growth factor-β1
TIE	Tyrosine kinase with immunoglobulin-like and EGF-like domains
TME	Tumor microenvironment
TNFα	Tumor necrosis factor α
VEGF(R)	Vascular endothelial growth factor (receptor)
VM	Vasculogenic mimicry
WHO	World Health Organization
Wnt	Wingless-related integration site
YAP	Yes-associated protein
YKL-40	Human cartilage glycoprotein HC-gp39, Chitinase-3-like protein 1, CHI3L1
ZEB	Zinc finger E-box-binding homeobox

## References

[1] Remon J., Pardo N., Martinez-Marti A., Cedres S., Navarro A., Martinez de Castro A.M., Felip E. (2017). Immune-checkpoint inhibition in first-line treatment of advanced non-small cell lung cancer patients: Current status and future approaches. Lung Cancer.

[2] Force J., Salama A.K. (2017). First-line treatment of metastatic melanoma: Role of nivolumab. Immunotargets Ther..

[3] Gajewski T.F., Schreiber H., Fu Y.X. (2013). Innate and adaptive immune cells in the tumor microenvironment. Nat. Immunol..

[4] Nakazawa M.S., Keith B., Simon M.C. (2016). Oxygen availability and metabolic adaptations. Nat. Rev. Cancer.

[5] Yeung S.J., Pan J., Lee M.H. (2008). Roles of p53, MYC and HIF-1 in regulating glycolysis—The seventh hallmark of cancer. Cell. Mol. Life Sci..

[6] Li Z., Zhang H. (2016). Reprogramming of glucose, fatty acid and amino acid metabolism for cancer progression. Cell. Mol. Life Sci..

[7] Lee N., Kim D. (2016). Cancer metabolism: Fueling more than just growth. Mol. Cells.

[8] Martinez-Outschoorn U.E., Peiris-Pages M., Pestell R.G., Sotgia F., Lisanti M.P. (2017). Cancer metabolism: A therapeutic perspective. Nat. Rev. Clin. Oncol..

[9] Hanahan D., Weinberg R.A. (2011). Hallmarks of cancer: The next generation. Cell.

[10] Yu L., Chen X., Wang L., Chen S. (2016). The sweet trap in tumors: Aerobic glycolysis and potential targets for therapy. Oncotarget.

[11] Dayton T.L., Jacks T., Vander Heiden M.G. (2016). PKM2, cancer metabolism, and the road ahead. EMBO Rep..

[12] Dong G., Mao Q., Xia W., Xu Y., Wang J., Xu L., Jiang F. (2016). PKM2 and cancer: The function of PKM2 beyond glycolysis (Review). Oncol. Lett..

[13] Li C., Zhang G., Zhao L., Ma Z., Chen H. (2016). Metabolic reprogramming in cancer cells: Glycolysis, glutaminolysis, and Bcl-2 proteins as novel therapeutic targets for cancer. World J. Surg. Oncol..

[14] Lee M., Yoon J.H. (2015). Metabolic interplay between glycolysis and mitochondrial oxidation: The reverse Warburg effect and its therapeutic implication. World J. Biol. Chem..

[15] Corbet C., Feron O. (2017). Cancer cell metabolism and mitochondria: Nutrient plasticity for TCA cycle fueling. Biochim. Biophys. Acta.

[16] De Vitto H., Perez-Valencia J., Radosevich J.A. (2016). Glutamine at focus: Versatile roles in cancer. Tumor Biol..

[17] Dang L., White D.W., Gross S., Bennett B.D., Bittinger M.A., Driggers E.M., Fantin V.R., Jang H.G., Jin S., Keenan M.C. (2009). Cancer-associated IDH1 mutations produce 2-hydroxyglutarate. Nature.

[18] Jin L., Alesi G.N., Kang S. (2016). Glutaminolysis as a target for cancer therapy. Oncogene.

[19] Hanahan D., Coussens L.M. (2012). Accessories to the crime: Functions of cells recruited to the tumor microenvironment. Cancer Cell.

[20] Martinez-Outschoorn U.E., Lisanti M.P., Sotgia F. (2014). Catabolic cancer-associated fibroblasts transfer energy and biomass to anabolic cancer cells, fueling tumor growth. Semin. Cancer Biol..

[21] Martinez-Outschoorn U.E., Sotgia F., Lisanti M.P. (2014). Metabolic asymmetry in cancer: A “balancing act” that promotes tumor growth. Cancer Cell.

[22] Rattigan Y.I., Patel B.B., Ackerstaff E., Sukenick G., Koutcher J.A., Glod J.W., Banerjee D. (2012). Lactate is a mediator of metabolic cooperation between stromal carcinoma associated fibroblasts and glycolytic tumor cells in the tumor microenvironment. Exp. Cell Res..

[23] Sotgia F., Martinez-Outschoorn U.E., Howell A., Pestell R.G., Pavlides S., Lisanti M.P. (2012). Caveolin-1 and cancer metabolism in the tumor microenvironment: Markers, models, and mechanisms. Annu. Rev. Pathol..

[24] Xing Y., Zhao S., Zhou B.P., Mi J. (2015). Metabolic reprogramming of the tumour microenvironment. FEBS J..

[25] Potente M., Carmeliet P. (2017). The Link Between Angiogenesis and Endothelial Metabolism. Annu. Rev. Physiol..

[26] Cantelmo A.R., Pircher A., Kalucka J., Carmeliet P. (2017). Vessel pruning or healing: Endothelial metabolism as a novel target?. Expert Opin. Ther. Targets.

[27] Behrens J. (1999). Cadherins and catenins: Role in signal transduction and tumor progression. Cancer Metastasis Rev..

[28] Le Bras G.F., Taubenslag K.J., Andl C.D. (2012). The regulation of cell-cell adhesion during epithelial-mesenchymal transition, motility and tumor progression. Cell Adhes. Migr..

[29] Gehler S., Ponik S.M., Riching K.M., Keely P.J. (2013). Bi-directional signaling: Extracellular matrix and integrin regulation of breast tumor progression. Crit. Rev. Eukaryot. Gene Expr..

[30] Xiong J., Balcioglu H.E., Danen E.H. (2013). Integrin signaling in control of tumor growth and progression. Int. J. Biochem. Cell Biol..

[31] Murphy G., Nagase H. (2011). Localizing matrix metalloproteinase activities in the pericellular environment. FEBS J..

[32] Papageorgis P., Stylianopoulos T. (2015). Role of TGFβ in regulation of the tumor microenvironment and drug delivery (Review). Int. J. Oncol..

[33] Renema N., Navet B., Heymann M.F., Lezot F., Heymann D. (2016). RANK-RANKL signalling in cancer. Biosci. Rep..

[34] Matsumoto K., Umitsu M., De Silva D.M., Roy A., Bottaro D.P. (2017). Hepatocyte growth factor/MET in cancer progression and biomarker discovery. Cancer Sci..

[35] Katoh M. (2016). FGFR inhibitors: Effects on cancer cells, tumor microenvironment and whole-body homeostasis (Review). Int. J. Mol. Med..

[36] Khan Z., Marshall J.F. (2016). The role of integrins in TGFβ activation in the tumour stroma. Cell Tissue Res..

[37] Goel H.L., Mercurio A.M. (2012). Enhancing integrin function by VEGF/neuropilin signaling: Implications for tumor biology. Cell Adhes. Migr..

[38] Jeanes A., Gottardi C.J., Yap A.S. (2008). Cadherins and cancer: How does cadherin dysfunction promote tumor progression?. Oncogene.

[39] Zhang H.G., Grizzle W.E. (2011). Exosomes and cancer: A newly described pathway of immune suppression. Clin. Cancer Res..

[40] Henderson M.C., Azorsa D.O. (2012). The genomic and proteomic content of cancer cell-derived exosomes. Front. Oncol..

[41] Dinger M.E., Mercer T.R., Mattick J.S. (2008). RNAs as extracellular signaling molecules. J. Mol. Endocrinol..

[42] Rashed M.H., Bayraktar E., Helel G.K., Abd-Ellah M.F., Amero P., Chavez-Reyes A., Rodriguez-Aguayo C. (2017). Exosomes: From garbage bins to promising therapeutic targets. Int. J. Mol. Sci..

[43] Thery C., Ostrowski M., Segura E. (2009). Membrane vesicles as conveyors of immune responses. Nat. Rev. Immunol..

[44] Paget S. (1989). The distribution of secondary growths in cancer of the breast. Cancer Metastasis Rev..

[45] Pietila M., Ivaska J., Mani S.A. (2016). Whom to blame for metastasis, the epithelial-mesenchymal transition or the tumor microenvironment?. Cancer Lett..

[46] Heldin C.H., Rubin K., Pietras K., Ostman A. (2004). High interstitial fluid pressure—An obstacle in cancer therapy. Nat. Rev. Cancer.

[47] Quail D.F., Joyce J.A. (2013). Microenvironmental regulation of tumor progression and metastasis. Nat. Med..

[48] Junttila M.R., de Sauvage F.J. (2013). Influence of tumour micro-environment heterogeneity on therapeutic response. Nature.

[49] Butcher D.T., Alliston T., Weaver V.M. (2009). A tense situation: Forcing tumour progression. Nat. Rev. Cancer.

[50] Fujii S., Fujihara A., Natori K., Abe A., Kuboki Y., Higuchi Y., Aizawa M., Kuwata T., Kinoshita T., Yasui W. (2015). TEM1 expression in cancer-associated fibroblasts is correlated with a poor prognosis in patients with gastric cancer. Cancer Med..

[51] Herrera M., Islam A.B., Herrera A., Martin P., Garcia V., Silva J., Garcia J.M., Salas C., Casal I., de Herreros A.G. (2013). Functional heterogeneity of cancer-associated fibroblasts from human colon tumors shows specific prognostic gene expression signature. Clin. Cancer Res..

[52] Yamashita M., Ogawa T., Zhang X., Hanamura N., Kashikura Y., Takamura M., Yoneda M., Shiraishi T. (2012). Role of stromal myofibroblasts in invasive breast cancer: Stromal expression of alpha-smooth muscle actin correlates with worse clinical outcome. Breast Cancer.

[53] Fujita H., Ohuchida K., Mizumoto K., Nakata K., Yu J., Kayashima T., Cui L., Manabe T., Ohtsuka T., Tanaka M. (2010). alpha-Smooth muscle actin expressing stroma promotes an aggressive tumor biology in pancreatic ductal adenocarcinoma. Pancreas.

[54] Mueller M.M., Fusenig N.E. (2004). Friends or foes—Bipolar effects of the tumour stroma in cancer. Nat. Rev. Cancer.

[55] Mattey D.L., Dawes P.T., Nixon N.B., Slater H. (1997). Transforming growth factor b1 and interleukin 4 induced a smooth muscle actin expression and myofibroblast-like differentiation in human synovial fibroblasts in vitro: Modulation by basic fibroblast growth factor. Ann. Rheum. Dis..

[56] Kalluri R. (2016). The biology and function of fibroblasts in cancer. Nat. Rev. Cancer.

[57] Micallef L., Vedrenne N., Billet F., Coulomb B., Darby I.A., Desmouliere A. (2012). The myofibroblast, multiple origins for major roles in normal and pathological tissue repair. Fibrogenes. Tissue Repair.

[58] Tomasek J.J., Gabbiani G., Hinz B., Chaponnier C., Brown R.A. (2002). Myofibroblasts and mechano-regulation of connective tissue remodelling. Nat. Rev. Mol. Cell Biol..

[59] Bhowmick N.A., Neilson E.G., Moses H.L. (2004). Stromal fibroblasts in cancer initiation and progression. Nature.

[60] Polanska U.M., Orimo A. (2013). Carcinoma-associated fibroblasts: Non-neoplastic tumour-promoting mesenchymal cells. J. Cell Physiol..

[61] Marsh T., Pietras K., McAllister S.S. (2013). Fibroblasts as architects of cancer pathogenesis. Biochim. Biophys. Acta.

[62] Kalluri R., Zeisberg M. (2006). Fibroblasts in cancer. Nat. Rev. Cancer.

[63] Webber J., Steadman R., Mason M.D., Tabi Z., Clayton A. (2010). Cancer exosomes trigger fibroblast to myofibroblast differentiation. Cancer Res..

[64] Dotto G.P., Weinberg R.A., Ariza A. (1988). Malignant transformation of mouse primary keratinocytes by Harvey sarcoma virus and its modulation by surrounding normal cells. Proc. Natl. Acad. Sci. USA.

[65] Serini G., Gabbiani G. (1999). Mechanisms of myofibroblast activity and phenotypic modulation. Exp. Cell Res..

[66] Strutz F., Okada H., Lo C.W., Danoff T., Carone R.L., Tomaszewski J.E., Neilson E.G. (1995). Identification and characterization of a fibroblast marker: FSP1. J. Cell Biol..

[67] Sugimoto H., Mundel T.M., Kieran M.W., Kalluri R. (2006). Identification of fibroblast heterogeneity in the tumor microenvironment. Cancer Biol. Ther..

[68] Garin-Chesa P., Old L.J., Rettig W.J. (1990). Cell surface glycoprotein of reactive stromal fibroblasts as a potential antibody target in human epithelial cancers. Proc. Natl. Acad. Sci. USA.

[69] Ni W.D., Yang Z.T., Cui C.A., Cui Y., Fang L.Y., Xuan Y.H. (2017). Tenascin-C is a potential cancer-associated fibroblasts marker and predicts poor prognosis in prostate cancer. Biochem. Biophys. Res. Commun..

[70] Orimo A., Weinberg R.A. (2006). Stromal fibroblasts in cancer: A novel tumor-promoting cell type. Cell Cycle.

[71] Paunescu V., Bojin F.M., Tatu C.A., Gavriliuc O.I., Rosca A., Gruia A.T., Tanasie G., Bunu C., Crisnic D., Gherghiceanu M. (2011). Tumour-associated fibroblasts and mesenchymal stem cells: More similarities than differences. J. Cell. Mol. Med..

[72] Micke P., Ostman A. (2004). Tumour-stroma interaction: Cancer-associated fibroblasts as novel targets in anti-cancer therapy?. Lung Cancer.

[73] Mueller L., Goumas F.A., Affeldt M., Sandtner S., Gehling U.M., Brilloff S., Walter J., Karnatz N., Lamszus K., Rogiers X. (2007). Stromal fibroblasts in colorectal liver metastases originate from resident fibroblasts and generate an inflammatory microenvironment. Am. J. Pathol..

[74] Zeisberg E.M., Potenta S., Xie L., Zeisberg M., Kalluri R. (2007). Discovery of endothelial to mesenchymal transition as a source for carcinoma-associated fibroblasts. Cancer Res..

[75] Yu Y., Xiao C.H., Tan L.D., Wang Q.S., Li X.Q., Feng Y.M. (2014). Cancer-associated fibroblasts induce epithelial-mesenchymal transition of breast cancer cells through paracrine TGF-β signalling. Br. J. Cancer.

[76] Fukumura D., Xavier R., Sugiura T., Chen Y., Park E.C., Lu N., Selig M., Nielsen G., Taksir T., Jain R.K. (1998). Tumor induction of VEGF promoter activity in stromal cells. Cell.

[77] Pietras K., Pahler J., Bergers G., Hanahan D. (2008). Functions of paracrine PDGF signaling in the proangiogenic tumor stroma revealed by pharmacological targeting. PLoS Med..

[78] Orimo A., Gupta P.B., Sgroi D.C., Arenzana-Seisdedos F., Delaunay T., Naeem R., Carey V.J., Richardson A.L., Weinberg R.A. (2005). Stromal fibroblasts present in invasive human breast carcinomas promote tumor growth and angiogenesis through elevated SDF-1/CXCL12 secretion. Cell.

[79] Suratt B.T., Petty J.M., Young S.K., Malcolm K.C., Lieber J.G., Nick J.A., Gonzalo J.A., Henson P.M., Worthen G.S. (2004). Role of the CXCR4/SDF-1 chemokine axis in circulating neutrophil homeostasis. Blood.

[80] Yang L., DeBusk L.M., Fukuda K., Fingleton B., Green-Jarvis B., Shyr Y., Matrisian L.M., Carbone D.P., Lin P.C. (2004). Expansion of myeloid immune suppressor Gr+CD11b+ cells in tumor-bearing host directly promotes tumor angiogenesis. Cancer Cell.

[81] Bergers G., Brekken R., McMahon G., Vu T.H., Itoh T., Tamaki K., Tanzawa K., Thorpe P., Itohara S., Werb Z. (2000). Matrix metalloproteinase-9 triggers the angiogenic switch during carcinogenesis. Nat. Cell Biol..

[82] Luga V., Zhang L., Viloria-Petit A.M., Ogunjimi A.A., Inanlou M.R., Chiu E., Buchanan M., Hosein A.N., Basik M., Wrana J.L. (2012). Exosomes mediate stromal mobilization of autocrine Wnt-PCP signaling in breast cancer cell migration. Cell.

[83] Gkretsi V., Stylianou A., Papageorgis P., Polydorou C., Stylianopoulos T. (2015). Remodeling components of the tumor microenvironment to enhance cancer therapy. Front. Oncol..

[84] Torimura T., Ueno T., Inuzuka S., Kin M., Ohira H., Kimura Y., Majima Y., Sata M., Abe H., Tanikawa K. (1994). The extracellular matrix in hepatocellular carcinoma shows different localization patterns depending on the differentiation and the histological pattern of tumors: Immunohistochemical analysis. J. Hepatol..

[85] Miyazaki K. (2006). Laminin-5 (laminin-332): Unique biological activity and role in tumor growth and invasion. Cancer Sci..

[86] Giannelli G., Bergamini C., Fransvea E., Sgarra C., Antonaci S. (2005). Laminin-5 with transforming growth factor-beta1 induces epithelial to mesenchymal transition in hepatocellular carcinoma. Gastroenterology.

[87] Ross J.B., Huh D., Noble L.B., Tavazoie S.F. (2015). Identification of molecular determinants of primary and metastatic tumour re-initiation in breast cancer. Nat. Cell Biol..

[88] Ghigna C., Valacca C., Biamonti G. (2008). Alternative splicing and tumor progression. Curr. Genom..

[89] Scarpino S., Stoppacciaro A., Pellegrini C., Marzullo A., Zardi L., Tartaglia F., Viale G., Ruco L.P. (1999). Expression of EDA/EDB isoforms of fibronectin in papillary carcinoma of the thyroid. J. Pathol..

[90] Hauptmann S., Zardi L., Siri A., Carnemolla B., Borsi L., Castellucci M., Klosterhalfen B., Hartung P., Weis J., Stocker G. (1995). Extracellular matrix proteins in colorectal carcinomas. Expression of tenascin and fibronectin isoforms. Lab. Investig..

[91] Bordeleau F., Califano J.P., Negron Abril Y.L., Mason B.N., LaValley D.J., Shin S.J., Weiss R.S., Reinhart-King C.A. (2015). Tissue stiffness regulates serine/arginine-rich protein-mediated splicing of the extra domain B-fibronectin isoform in tumors. Proc. Natl. Acad. Sci. USA.

[92] Serini G., Bochaton-Piallat M.L., Ropraz P., Geinoz A., Borsi L., Zardi L., Gabbiani G. (1998). The fibronectin domain ED-A is crucial for myofibroblastic phenotype induction by transforming growth factor-beta1. J. Cell Biol..

[93] Kuhn C., McDonald J.A. (1991). The roles of the myofibroblast in idiopathic pulmonary fibrosis. Ultrastructural and immunohistochemical features of sites of active extracellular matrix synthesis. Am. J. Pathol..

[94] Han Z., Zhou Z., Shi X., Wang J., Wu X., Sun D., Chen Y., Zhu H., Magi-Galluzzi C., Lu Z.R. (2015). EDB Fibronectin specific peptide for prostate cancer targeting. Bioconjug. Chem..

[95] Oskarsson T., Massague J. (2012). Extracellular matrix players in metastatic niches. EMBO J..

[96] Malanchi I., Santamaria-Martinez A., Susanto E., Peng H., Lehr H.A., Delaloye J.F., Huelsken J. (2011). Interactions between cancer stem cells and their niche govern metastatic colonization. Nature.

[97] Oskarsson T., Acharyya S., Zhang X.H., Vanharanta S., Tavazoie S.F., Morris P.G., Downey R.J., Manova-Todorova K., Brogi E., Massague J. (2011). Breast cancer cells produce tenascin C as a metastatic niche component to colonize the lungs. Nat. Med..

[98] Egbert M., Ruetze M., Sattler M., Wenck H., Gallinat S., Lucius R., Weise J.M. (2014). The matricellular protein periostin contributes to proper collagen function and is downregulated during skin aging. J. Dermatol. Sci..

[99] Kii I., Nishiyama T., Li M., Matsumoto K., Saito M., Amizuka N., Kudo A. (2010). Incorporation of tenascin-C into the extracellular matrix by periostin underlies an extracellular meshwork architecture. J. Biol. Chem..

[100] Degen M., Brellier F., Kain R., Ruiz C., Terracciano L., Orend G., Chiquet-Ehrismann R. (2007). Tenascin-W is a novel marker for activated tumor stroma in low-grade human breast cancer and influences cell behavior. Cancer Res..

[101] Brellier F., Tucker R.P., Chiquet-Ehrismann R. (2009). Tenascins and their implications in diseases and tissue mechanics. Scand. J. Med. Sci. Sports.

[102] Degen M., Brellier F., Schenk S., Driscoll R., Zaman K., Stupp R., Tornillo L., Terracciano L., Chiquet-Ehrismann R., Ruegg C. (2008). Tenascin-W, a new marker of cancer stroma, is elevated in sera of colon and breast cancer patients. Int. J. Cancer.

[103] Scherberich A., Tucker R.P., Degen M., Brown-Luedi M., Andres A.C., Chiquet-Ehrismann R. (2005). Tenascin-W is found in malignant mammary tumors, promotes alpha8 integrin-dependent motility and requires p38MAPK activity for BMP-2 and TNF-alpha induced expression in vitro. Oncogene.

[104] Akiri G., Sabo E., Dafni H., Vadasz Z., Kartvelishvily Y., Gan N., Kessler O., Cohen T., Resnick M., Neeman M. (2003). Lysyl oxidase-related protein-1 promotes tumor fibrosis and tumor progression in vivo. Cancer Res..

[105] Smith-Mungo L.I., Kagan H.M. (1998). Lysyl oxidase: Properties, regulation and multiple functions in biology. Matrix Biol..

[106] Paszek M.J., Zahir N., Johnson K.R., Lakins J.N., Rozenberg G.I., Gefen A., Reinhart-King C.A., Margulies S.S., Dembo M., Boettiger D. (2005). Tensional homeostasis and the malignant phenotype. Cancer Cell.

[107] Weaver V.M., Lelievre S., Lakins J.N., Chrenek M.A., Jones J.C., Giancotti F., Werb Z., Bissell M.J. (2002). b4 integrin-dependent formation of polarized three-dimensional architecture confers resistance to apoptosis in normal and malignant mammary epithelium. Cancer Cell.

[108] White D.E., Kurpios N.A., Zuo D., Hassell J.A., Blaess S., Mueller U., Muller W.J. (2004). Targeted disruption of b1-integrin in a transgenic mouse model of human breast cancer reveals an essential role in mammary tumor induction. Cancer Cell.

[109] Wipff P.J., Rifkin D.B., Meister J.J., Hinz B. (2007). Myofibroblast contraction activates latent TGF-β1 from the extracellular matrix. J. Cell Biol..

[110] Calvo F., Ege N., Grande-Garcia A., Hooper S., Jenkins R.P., Chaudhry S.I., Harrington K., Williamson P., Moeendarbary E., Charras G. (2013). Mechanotransduction and YAP-dependent matrix remodelling is required for the generation and maintenance of cancer-associated fibroblasts. Nat. Cell Biol..

[111] Sahai E., Marshall C.J. (2002). ROCK and Dia have opposing effects on adherens junctions downstream of Rho. Nat. Cell Biol..

[112] Burridge K., Wennerberg K. (2004). Rho and Rac take center stage. Cell.

[113] Fan Y., Shen B., Tan M., Mu X., Qin Y., Zhang F., Liu Y. (2014). TGF-β-induced upregulation of malat1 promotes bladder cancer metastasis by associating with suz12. Clin. Cancer Res..

[114] Jodele S., Blavier L., Yoon J.M., DeClerck Y.A. (2006). Modifying the soil to affect the seed: Role of stromal-derived matrix metalloproteinases in cancer progression. Cancer Metastasis Rev..

[115] Fang M., Yuan J., Peng C., Li Y. (2014). Collagen as a double-edged sword in tumor progression. Tumor Biol..

[116] Bergamaschi A., Tagliabue E., Sorlie T., Naume B., Triulzi T., Orlandi R., Russnes H.G., Nesland J.M., Tammi R., Auvinen P. (2008). Extracellular matrix signature identifies breast cancer subgroups with different clinical outcome. J. Pathol..

[117] Monboisse J.C., Oudart J.B., Ramont L., Brassart-Pasco S., Maquart F.X. (2014). Matrikines from basement membrane collagens: A new anti-cancer strategy. Biochim. Biophys. Acta.

[118] Folkman J. (2006). Antiangiogenesis in cancer therapy—Endostatin and its mechanisms of action. Exp. Cell Res..

[119] Willis C.D., Poluzzi C., Mongiat M., Iozzo R.V. (2013). Endorepellin laminin-like globular 1/2 domains bind Ig3–5 of vascular endothelial growth factor (VEGF) receptor 2 and block pro-angiogenic signaling by VEGFA in endothelial cells. FEBS J..

[120] Billioux A., Modlich U., Bicknell R., Alison M. (2007). Angiogenesis. The Cancer Handbook.

[121] Folkman J. (2002). Looking for a good endothelial address. Cancer Cell.

[122] Hanahan D., Folkman J. (1996). Patterns and emerging mechanisms of the angiogenic switch during tumorigenesis. Cell.

[123] Blouw B., Song H., Tihan T., Bosze J., Ferrara N., Gerber H.-P., Johnson R.S., Bergers G. (2003). The hypoxic response of tumors is dependent on their microenvironment. Cancer Cell.

[124] Folkman J., Watson K., Ingber D., Hanahan D. (1989). Induction of angiogenesis during the transition from hyperplasia to neoplasia. Nature.

[125] Weidner N., Semple J.P., Welch W.R., Folkman J. (1991). Tumor angiogenesis and metastasis—Correlation in invasive breast carcinoma. N. Engl. J. Med..

[126] Kandel J., Bossy-Wetzel E., Radvanyi F., Klagsbrun M., Folkman J., Hanahan D. (1991). Neovascularization is associated with a switch to the export of bFGF in the multistep development of fibrosarcoma. Cell.

[127] Niland S., Eble J.A. (2012). Integrin-mediated cell-matrix interaction in physiological and pathological blood vessel formation. J. Oncol..

[128] Sica A., Schioppa T., Mantovani A., Allavena P. (2006). Tumour-associated macrophages are a distinct M2 polarised population promoting tumour progression: Potential targets of anti-cancer therapy. Eur. J. Cancer.

[129] Lin E.Y., Pollard J.W. (2007). Tumor-associated macrophages press the angiogenic switch in breast cancer. Cancer Res..

[130] Schmid M.C., Varner J.A. (2007). Myeloid cell trafficking and tumor angiogenesis. Cancer Lett..

[131] Aplin A.C., Fogel E., Nicosia R.F. (2010). MCP-1 promotes mural cell recruitment during angiogenesis in the aortic ring model. Angiogenesis.

[132] Hong K.H., Ryu J., Han K.H. (2005). Monocyte chemoattractant protein-1-induced angiogenesis is mediated by vascular endothelial growth factor-A. Blood.

[133] Niu J., Azfer A., Zhelyabovska O., Fatma S., Kolattukudy P.E. (2008). Monocyte chemotactic protein (MCP)-1 promotes angiogenesis via a novel transcription factor, MCP-1-induced protein (MCPIP). J. Biol. Chem..

[134] Bergers G., Hanahan D. (2008). Modes of resistance to anti-angiogenic therapy. Nat. Rev. Cancer.

[135] Hanahan D., Weinberg R.A. (2000). The hallmarks of cancer. Cell.

[136] Nagy J.A., Chang S.H., Dvorak A.M., Dvorak H.F. (2009). Why are tumour blood vessels abnormal and why is it important to know?. Br. J. Cancer.

[137] Dome B., Hendrix M.J., Paku S., Tovari J., Timar J. (2007). Alternative vascularization mechanisms in cancer: Pathology and therapeutic implications. Am. J. Pathol..

[138] Hillen F., Griffioen A.W. (2007). Tumour vascularization: Sprouting angiogenesis and beyond. Cancer Metastasis Rev..

[139] Nagy J.A., Dvorak H.F. (2012). Heterogeneity of the tumor vasculature: The need for new tumor blood vessel type-specific targets. Clin. Exp. Metastasis.

[140] Kaspar M., Zardi L., Neri D. (2006). Fibronectin as target for tumor therapy. Int. J. Cancer.

[141] Midulla M., Verma R., Pignatelli M., Ritter M.A., Courtenay-Luck N.S., George A.J.T. (2000). Source of oncofetal ED-B-containing fibronectin: Implications of production by both tumor and endothelial cells. Cancer Res..

[142] Midwood K.S., Orend G. (2009). The role of tenascin-C in tissue injury and tumorigenesis. J. Cell Commun. Signal..

[143] Pezzolo A., Parodi F., Marimpietri D., Raffaghello L., Cocco C., Pistorio A., Mosconi M., Gambini C., Cilli M., Deaglio S. (2011). Oct-4+/Tenascin C+ neuroblastoma cells serve as progenitors of tumor-derived endothelial cells. Cell Res..

[144] Martina E., Chiquet-Ehrismann R., Brellier F. (2010). Tenascin-W: An extracellular matrix protein associated with osteogenesis and cancer. Int. J. Biochem. Cell Biol..

[145] Dudley A.C. (2012). Tumor endothelial cells. Cold Spring Harb. Perspect. Med..

[146] Benjamin L.E., Hemo I., Keshet E. (1998). A plasticity window for blood vessel remodelling is defined by pericyte coverage of the preformed endothelial network and is regulated by PDGF-B and VEGF. Development.

[147] Burgers A.C., Lammert E. (2011). Extraerythrocytic hemoglobin—A possible oxygen transporter in human malignant tumors. Med. Hypotheses.

[148] Maniotis A.J., Folberg R., Hess A., Seftor E.A., Gardner L.M., Pe’er J., Trent J.M., Meltzer P.S., Hendrix M.J. (1999). Vascular channel formation by human melanoma cells in vivo and in vitro: Vasculogenic mimicry. Am. J. Pathol..

[149] Sun B., Zhang D., Zhao N., Zhao X. (2017). Epithelial-to-endothelial transition and cancer stem cells: Two cornerstones of vasculogenic mimicry in malignant tumors. Oncotarget.

[150] Dvorak H.F., Senger D.R., Dvorak A.M. (1983). Fibrin as a component of the tumor stroma: Origins and biological significance. Cancer Metastasis Rev..

[151] Dvorak H.F. (1986). Tumors: Wounds that do not heal. Similarities between tumor stroma generation and wound healing. N. Engl. J. Med..

[152] Dvorak H.F., Brown L.F., Detmar M., Dvorak A.M. (1995). Vascular permeability factor/vascular endothelial growth factor, microvascular hyperpermeability, and angiogenesis. Am. J. Pathol..

[153] Jain R.K. (2001). Normalizing tumor vasculature with anti-angiogenic therapy: A new paradigm for combination therapy. Nat. Med..

[154] Holash J., Maisonpierre P.C., Compton D., Boland P., Alexander C.R., Zagzag D., Yancopoulos G.D., Wiegand S.J. (1999). Vessel cooption, regression, and growth in tumors mediated by angiopoietins and VEGF. Science.

[155] Donnem T., Hu J., Ferguson M., Adighibe O., Snell C., Harris A.L., Gatter K.C., Pezzella F. (2013). Vessel co-option in primary human tumors and metastases: An obstacle to effective anti-angiogenic treatment?. Cancer Med..

[156] Asahara T., Murohara T., Sullivan A., Silver M., van der Zee R., Li T., Witzenbichler B., Schatteman G., Isner J.M. (1997). Isolation of putative progenitor endothelial cells for angiogenesis. Science.

[157] Ruoslahti E. (2002). Specialization of tumour vasculature. Nat. Rev. Cancer.

[158] Potente M., Gerhardt H., Carmeliet P. (2011). Basic and therapeutic aspects of angiogenesis. Cell.

[159] Carmeliet P. (2003). Angiogenesis in health and disease. Nat. Med..

[160] Iruela-Arispe M.L., Davis G.E. (2009). Cellular and molecular mechanisms of vascular lumen formation. Dev. Cell.

[161] Strilic B., Kucera T., Eglinger J., Hughes M.R., McNagny K.M., Tsukita S., Dejana E., Ferrara N., Lammert E. (2009). The molecular basis of vascular lumen formation in the developing mouse aorta. Dev. Cell.

[162] Zovein A.C., Luque A., Turlo K.A., Hofmann J.J., Yee K.M., Becker M.S., Fassler R., Mellman I., Lane T.F., Iruela-Arispe M.L. (2010). Beta1 integrin establishes endothelial cell polarity and arteriolar lumen formation via a Par3-dependent mechanism. Dev. Cell.

[163] Djonov V., Schmid M., Tschanz S.A., Burri P.H. (2000). Intussusceptive angiogenesis: Its role in embryonic vascular network formation. Circ. Res..

[164] Kurz H., Burri P.H., Djonov V.G. (2003). Angiogenesis and vascular remodeling by intussusception: From form to function. News Physiol. Sci..

[165] Gianni-Barrera R., Trani M., Reginato S., Banfi A. (2011). To sprout or to split? VEGF, Notch and vascular morphogenesis. Biochem. Soc. Trans..

[166] Nico B., Crivellato E., Guidolin D., Annese T., Longo V., Finato N., Vacca A., Ribatti D. (2010). Intussusceptive microvascular growth in human glioma. Clin. Exp. Med..

[167] Brat D.J., Van Meir E.G. (2001). Glomeruloid microvascular proliferation orchestrated by VPF/VEGF: A new world of angiogenesis research. Am. J. Pathol..

[168] Straume O., Chappuis P.O., Salvesen H.B., Halvorsen O.J., Haukaas S.A., Goffin J.R., Begin L.R., Foulkes W.D., Akslen L.A. (2002). Prognostic importance of glomeruloid microvascular proliferation indicates an aggressive angiogenic phenotype in human cancers. Cancer Res..

[169] Lyden D., Hattori K., Dias S., Costa C., Blaikie P., Butros L., Chadburn A., Heissig B., Marks W., Witte L. (2001). Impaired recruitment of bone-marrow-derived endothelial and hematopoietic precursor cells blocks tumor angiogenesis and growth. Nat. Med..

[170] Reyes M., Dudek A., Jahagirdar B., Koodie L., Marker P.H., Verfaillie C.M. (2002). Origin of endothelial progenitors in human postnatal bone marrow. J. Clin. Investig..

[171] Ribatti D. (2004). The involvement of endothelial progenitor cells in tumor angiogenesis. J. Cell. Mol. Med..

[172] Moschetta M., Mishima Y., Sahin I., Manier S., Glavey S., Vacca A., Roccaro A.M., Ghobrial I.M. (2014). Role of endothelial progenitor cells in cancer progression. Biochim. Biophys. Acta.

[173] Ricci-Vitiani L., Pallini R., Biffoni M., Todaro M., Invernici G., Cenci T., Maira G., Parati E.A., Stassi G., Larocca L.M. (2010). Tumour vascularization via endothelial differentiation of glioblastoma stem-like cells. Nature.

[174] Kirschmann D.A., Seftor E.A., Hardy K.M., Seftor R.E., Hendrix M.J. (2012). Molecular pathways: Vasculogenic mimicry in tumor cells: Diagnostic and therapeutic implications. Clin. Cancer Res..

[175] Folberg R., Maniotis A.J. (2004). Vasculogenic mimicry. APMIS.

[176] Lin A.Y., Maniotis A.J., Valyi-Nagy K., Majumdar D., Setty S., Kadkol S., Leach L., Pe’er J., Folberg R. (2005). Distinguishing Fibrovascular Septa From Vasculogenic Mimicry Patterns. Arch. Pathol. Lab. Med..

[177] El Hallani S., Boisselier B., Peglion F., Rousseau A., Colin C., Idbaih A., Marie Y., Mokhtari K., Thomas J.L., Eichmann A. (2010). A new alternative mechanism in glioblastoma vascularization: Tubular vasculogenic mimicry. Brain.

[178] Bajcsy P., Lee S.C., Lin A., Folberg R. (2006). Three-dimensional volume reconstruction of extracellular matrix proteins in uveal melanoma from fluorescent confocal laser scanning microscope images. J. Microsc..

[179] Clarijs R., Otte-Holler I., Ruiter D.J., de Waal R.M. (2002). Presence of a fluid-conducting meshwork in xenografted cutaneous and primary human uveal melanoma. Investig. Ophthalmol. Vis. Sci..

[180] Ahn G.O., Brown J.M. (2008). Matrix metalloproteinase-9 is required for tumor vasculogenesis but not for angiogenesis: Role of bone marrow-derived myelomonocytic cells. Cancer Cell.

[181] Iivanainen E., Kahari V.M., Heino J., Elenius K. (2003). Endothelial cell-matrix interactions. Microsc. Res. Tech..

[182] Rundhaug J.E. (2005). Matrix metalloproteinases and angiogenesis. J. Cell. Mol. Med..

[183] Eble J.A., Niland S. (2009). The extracellular matrix of blood vessels. Curr. Pharm. Des..

[184] Andaloussi S.E.L., Mager I., Breakefield X.O., Wood M.J. (2013). Extracellular vesicles: Biology and emerging therapeutic opportunities. Nat. Rev. Drug Discov..

[185] Tirziu D., Giordano F.J., Simons M. (2010). Cell communications in the heart. Circulation.

[186] Kedrin D., Gligorijevic B., Wyckoff J., Verkhusha V.V., Condeelis J., Segall J.E., van Rheenen J. (2008). Intravital imaging of metastatic behavior through a mammary imaging window. Nat. Methods.

[187] Mierke C.T., Zitterbart D.P., Kollmannsberger P., Raupach C., Schlotzer-Schrehardt U., Goecke T.W., Behrens J., Fabry B. (2008). Breakdown of the endothelial barrier function in tumor cell transmigration. Biophys. J..

[188] Olsson A.K., Dimberg A., Kreuger J., Claesson-Welsh L. (2006). VEGF receptor signalling—In control of vascular function. Nat. Rev. Mol. Cell Biol..

[189] Weis S.M., Cheresh D.A. (2011). alphaV integrins in angiogenesis and cancer. Cold Spring Harb. Perspect. Med..

[190] Dvorak H.F. (2002). Vascular permeability factor/vascular endothelial growth factor: A critical cytokine in tumor angiogenesis and a potential target for diagnosis and therapy. J. Clin. Oncol..

[191] Guo F., Wang Y., Liu J., Mok S.C., Xue F., Zhang W. (2016). CXCL12/CXCR4: A symbiotic bridge linking cancer cells and their stromal neighbors in oncogenic communication networks. Oncogene.

[192] Teicher B.A., Fricker S.P. (2010). CXCL12 (SDF-1)/CXCR4 pathway in cancer. Clin. Cancer Res..

[193] Reymond N., d’Agua B.B., Ridley A.J. (2013). Crossing the endothelial barrier during metastasis. Nat. Rev. Cancer.

[194] Liu F., Lang R., Wei J., Fan Y., Cui L., Gu F., Guo X., Pringle G.A., Zhang X., Fu L. (2009). Increased expression of SDF-1/CXCR4 is associated with lymph node metastasis of invasive micropapillary carcinoma of the breast. Histopathology.

[195] Iwasa S., Yanagawa T., Fan J., Katoh R. (2009). Expression of CXCR4 and its ligand SDF-1 in intestinal-type gastric cancer is associated with lymph node and liver metastasis. Anticancer Res..

[196] Liang J.J., Zhu S., Bruggeman R., Zaino R.J., Evans D.B., Fleming J.B., Gomez H.F., Zander D.S., Wang H. (2010). High levels of expression of human stromal cell-derived factor-1 are associated with worse prognosis in patients with stage II pancreatic ductal adenocarcinoma. Cancer Epidemiol. Biomark. Prev..

[197] Thomas R.M., Kim J., Revelo-Penafiel M.P., Angel R., Dawson D.W., Lowy A.M. (2008). The chemokine receptor CXCR4 is expressed in pancreatic intraepithelial neoplasia. Gut.

[198] Guo L., Cui Z.M., Zhang J., Huang Y. (2011). Chemokine axes CXCL12/CXCR4 and CXCL16/CXCR6 correlate with lymph node metastasis in epithelial ovarian carcinoma. Chin. J. Cancer.

[199] Yu Y., Shi X., Shu Z., Xie T., Huang K., Wei L., Song H., Zhang W., Xue X. (2013). Stromal cell-derived factor-1 (SDF-1)/CXCR4 axis enhances cellular invasion in ovarian carcinoma cells via integrin beta1 and beta3 expressions. Oncol. Res..

[200] Huang Y., Zhang J., Cui Z.M., Zhao J., Zheng Y. (2013). Expression of the CXCL12/CXCR4 and CXCL16/CXCR6 axes in cervical intraepithelial neoplasia and cervical cancer. Chin. J. Cancer.

[201] Murakami T., Maki W., Cardones A.R., Fang H., Tun Kyi A., Nestle F.O., Hwang S.T. (2002). Expression of CXC chemokine receptor-4 enhances the pulmonary metastatic potential of murine B16 melanoma cells. Cancer Res..

[202] Hayes J., Peruzzi P.P., Lawler S. (2014). MicroRNAs in cancer: Biomarkers, functions and therapy. Trends Mol. Med..

[203] Kohlhapp F.J., Mitra A.K., Lengyel E., Peter M.E. (2015). MicroRNAs as mediators and communicators between cancer cells and the tumor microenvironment. Oncogene.

[204] Wurdinger T., Tannous B.A., Saydam O., Skog J., Grau S., Soutschek J., Weissleder R., Breakefield X.O., Krichevsky A.M. (2008). miR-296 regulates growth factor receptor overexpression in angiogenic endothelial cells. Cancer Cell.

[205] Boon R.A., Vickers K.C. (2013). Intercellular transport of microRNAs. Arterioscler Thromb Vasc. Biol..

[206] Zhuang G., Wu X., Jiang Z., Kasman I., Yao J., Guan Y., Oeh J., Modrusan Z., Bais C., Sampath D. (2012). Tumour-secreted miR-9 promotes endothelial cell migration and angiogenesis by activating the JAK-STAT pathway. EMBO J..

[207] Carmeliet P., Jain R.K. (2000). Angiogenesis in cancer and other diseases. Nature.

[208] Selek L., Dhobb M., van der Sanden B., Berger F., Wion D. (2011). Existence of tumor-derived endothelial cells suggests an additional role for endothelial-to-mesenchymal transition in tumor progression. Int. J. Cancer.

[209] Ghiabi P., Jiang J., Pasquier J., Maleki M., Abu-Kaoud N., Rafii S., Rafii A. (2014). Endothelial cells provide a notch-dependent pro-tumoral niche for enhancing breast cancer survival, stemness and pro-metastatic properties. PLoS ONE.

[210] Gregory L.A., Ricart R.A., Patel S.A., Lim P.K., Rameshwar P. (2011). microRNAs, Gap Junctional Intercellular Communication and Mesenchymal Stem Cells in Breast Cancer Metastasis. Curr. Cancer Ther. Rev..

[211] Valiunas V., Polosina Y.Y., Miller H., Potapova I.A., Valiuniene L., Doronin S., Mathias R.T., Robinson R.B., Rosen M.R., Cohen I.S. (2005). Connexin-specific cell-to-cell transfer of short interfering RNA by gap junctions. J. Physiol..

[212] Kizana E., Cingolani E., Marban E. (2009). Non-cell-autonomous effects of vector-expressed regulatory RNAs in mammalian heart cells. Gene Ther..

[213] Leithe E., Sirnes S., Omori Y., Rivedal E. (2006). Downregulation of gap junctions in cancer cells. Crit. Rev. Oncog..

[214] Lopes-Bastos B.M., Jiang W.G., Cai J. (2016). Tumour-Endothelial Cell Communications: Important and Indispensable Mediators of Tumour Angiogenesis. Anticancer Res..

[215] Pollmann M.A., Shao Q., Laird D.W., Sandig M. (2005). Connexin 43 mediated gap junctional communication enhances breast tumor cell diapedesis in culture. Breast Cancer Res..

[216] Elzarrad M.K., Haroon A., Willecke K., Dobrowolski R., Gillespie M.N., Al-Mehdi A.B. (2008). Connexin-43 upregulation in micrometastases and tumor vasculature and its role in tumor cell attachment to pulmonary endothelium. BMC Med..

[217] Ito A., Katoh F., Kataoka T.R., Okada M., Tsubota N., Asada H., Yoshikawa K., Maeda S., Kitamura Y., Yamasaki H. (2000). A role for heterologous gap junctions between melanoma and endothelial cells in metastasis. J. Clin. Investig..

[218] Cai J., Jiang W.G., Mansel R.E. (1998). Gap junctional communication and the tyrosine phosphorylation of connexin 43 in interaction between breast cancer and endothelial cells. Int. J. Mol. Med..

[219] Zibara K., Awada Z., Dib L., El-Saghir J., Al-Ghadban S., Ibrik A., El-Zein N., El-Sabban M. (2015). Anti-angiogenesis therapy and gap junction inhibition reduce MDA-MB-231 breast cancer cell invasion and metastasis in vitro and in vivo. Sci. Rep..

[220] Esser S., Lampugnani M.G., Corada M., Dejana E., Risau W. (1998). Vascular endothelial growth factor induces VE-cadherin tyrosine phosphorylation in endothelial cells. J. Cell Sci..

[221] Lampugnani M.G., Corada M., Caveda L., Breviario F., Ayalon O., Geiger B., Dejana E. (1995). The molecular organization of endothelial cell to cell junctions: Differential association of plakoglobin, beta-catenin, and alpha-catenin with vascular endothelial cadherin (VE-cadherin). J. Cell Biol..

[222] Lampugnani M.G., Resnati M., Raiteri M., Pigott R., Pisacane A., Houen G., Ruco L.P., Dejana E. (1992). A novel endothelial-specific membrane protein is a marker of cell-cell contacts. J. Cell Biol..

[223] Wallez Y., Vilgrain I., Huber P. (2006). Angiogenesis: The VE-cadherin switch. Trends Cardiovasc. Med..

[224] Liao F., Li Y., O’Connor W., Zanetta L., Bassi R., Santiago A., Overholser J., Hooper A., Mignatti P., Dejana E. (2000). Monoclonal antibody to vascular endothelial-cadherin is a potent inhibitor of angiogenesis, tumor growth, and metastasis. Cancer Res..

[225] Wessel F., Winderlich M., Holm M., Frye M., Rivera-Galdos R., Vockel M., Linnepe R., Ipe U., Stadtmann A., Zarbock A. (2014). Leukocyte extravasation and vascular permeability are each controlled in vivo by different tyrosine residues of VE-cadherin. Nat. Immunol..

[226] Gavard J. (2014). Endothelial permeability and VE-cadherin: A wacky comradeship. Cell Adhes. Migr..

[227] Potter M.D., Barbero S., Cheresh D.A. (2005). Tyrosine phosphorylation of VE-cadherin prevents binding of p120- and beta-catenin and maintains the cellular mesenchymal state. J. Biol. Chem..

[228] Peng H.H., Hodgson L., Henderson A.J., Dong C. (2005). Involvement of phospholipase C signaling in melanoma cell-induced endothelial junction disassembly. Front. Biosci..

[229] Haidari M., Zhang W., Caivano A., Chen Z., Ganjehei L., Mortazavi A., Stroud C., Woodside D.G., Willerson J.T., Dixon R.A. (2012). Integrin alpha2beta1 mediates tyrosine phosphorylation of vascular endothelial cadherin induced by invasive breast cancer cells. J. Biol. Chem..

[230] Aragon-Sanabria V., Pohler S.E., Eswar V.J., Bierowski M., Gomez E.W., Dong C. (2017). VE-Cadherin Disassembly and Cell Contractility in the Endothelium are Necessary for Barrier Disruption Induced by Tumor Cells. Sci. Rep..

[231] McDonald D.M., Munn L., Jain R.K. (2000). Vasculogenic mimicry: How convincing, how novel, and how significant?. Am. J. Pathol..

[232] Cao Z., Shang B., Zhang G., Miele L., Sarkar F.H., Wang Z., Zhou Q. (2013). Tumor cell-mediated neovascularization and lymphangiogenesis contrive tumor progression and cancer metastasis. Biochim. Biophys. Acta.

[233] Murphy G.F., Wilson B.J., Girouard S.D., Frank N.Y., Frank M.H. (2014). Stem cells and targeted approaches to melanoma cure. Mol. Asp. Med..

[234] Potgens A.J., van Altena M.C., Lubsen N.H., Ruiter D.J., de Waal R.M. (1996). Analysis of the tumor vasculature and metastatic behavior of xenografts of human melanoma cell lines transfected with vascular permeability factor. Am. J. Pathol..

[235] Lammert E., Axnick J. (2012). Vascular lumen formation. Cold Spring Harb. Perspect. Med..

[236] Kobayashi H., Shirakawa K., Kawamoto S., Saga T., Sato N., Hiraga A., Watanabe I., Heike Y., Togashi K., Konishi J. (2002). Rapid accumulation and internalization of radiolabeled herceptin in an inflammatory breast cancer xenograft with vasculogenic mimicry predicted by the contrast-enhanced dynamic MRI with the macromolecular contrast agent G6-(1B4M-Gd)(256). Cancer Res..

[237] Shirakawa K., Kobayashi H., Heike Y., Kawamoto S., Brechbiel M.W., Kasumi F., Iwanaga T., Konishi F., Terada M., Wakasugi H. (2002). Hemodynamics in vasculogenic mimicry and angiogenesis of inflammatory breast cancer xenograft. Cancer Res..

[238] Liu Z., Li Y., Zhao W., Ma Y., Yang X. (2011). Demonstration of vasculogenic mimicry in astrocytomas and effects of Endostar on U251 cells. Pathol. Res. Pract..

[239] Thies A., Mangold U., Moll I., Schumacher U. (2001). PAS-positive loops and networks as a prognostic indicator in cutaneous malignant melanoma. J. Pathol..

[240] Mihic-Probst D., Ikenberg K., Tinguely M., Schraml P., Behnke S., Seifert B., Civenni G., Sommer L., Moch H., Dummer R. (2012). Tumor cell plasticity and angiogenesis in human melanomas. PLoS ONE.

[241] Sun W., Fan Y.Z., Zhang W.Z., Ge C.Y. (2011). A pilot histomorphology and hemodynamic of vasculogenic mimicry in gallbladder carcinomas in vivo and in vitro. J. Exp. Clin. Cancer Res..

[242] Guo J.Q., Zheng Q.H., Chen H., Chen L., Xu J.B., Chen M.Y., Lu D., Wang Z.H., Tong H.F., Lin S. (2014). Ginsenoside Rg3 inhibition of vasculogenic mimicry in pancreatic cancer through downregulation of VEcadherin/EphA2/MMP9/MMP2 expression. Int. J. Oncol..

[243] Sun B., Zhang S., Zhang D., Du J., Guo H., Zhao X., Zhang W., Hao X. (2006). Vasculogenic mimicry is associated with high tumor grade, invasion and metastasis, and short survival in patients with hepatocellular carcinoma. Oncol. Rep..

[244] Tang N.N., Zhu H., Zhang H.J., Zhang W.F., Jin H.L., Wang L., Wang P., He G.J., Hao B., Shi R.H. (2014). HIF-1alpha induces VE-cadherin expression and modulates vasculogenic mimicry in esophageal carcinoma cells. World J. Gastroenterol..

[245] Sun B., Qie S., Zhang S., Sun T., Zhao X., Gao S., Ni C., Wang X., Liu Y., Zhang L. (2008). Role and mechanism of vasculogenic mimicry in gastrointestinal stromal tumors. Hum. Pathol..

[246] Baeten C.I., Hillen F., Pauwels P., de Bruine A.P., Baeten C.G. (2009). Prognostic role of vasculogenic mimicry in colorectal cancer. Dis. Colon Rectum.

[247] Williamson S.C., Metcalf R.L., Trapani F., Mohan S., Antonello J., Abbott B., Leong H.S., Chester C.P., Simms N., Polanski R. (2016). Vasculogenic mimicry in small cell lung cancer. Nat. Commun..

[248] Wu S., Yu L., Wang D., Zhou L., Cheng Z., Chai D., Ma L., Tao Y. (2012). Aberrant expression of CD133 in non-small cell lung cancer and its relationship to vasculogenic mimicry. BMC Cancer.

[249] Sood A.K., Seftor E.A., Fletcher M.S., Gardner L.M., Heidger P.M., Buller R.E., Seftor R.E., Hendrix M.J. (2001). Molecular determinants of ovarian cancer plasticity. Am. J. Pathol..

[250] Tang H.S., Feng Y.J., Yao L.Q. (2009). Angiogenesis, vasculogenesis, and vasculogenic mimicry in ovarian cancer. Int. J. Gynecol. Cancer.

[251] Sharma N., Seftor R.E., Seftor E.A., Gruman L.M., Heidger P.M., Cohen M.B., Lubaroff D.M., Hendrix M.J. (2002). Prostatic tumor cell plasticity involves cooperative interactions of distinct phenotypic subpopulations: Role in vasculogenic mimicry. Prostate.

[252] Cai X.S., Jia Y.W., Mei J., Tang R.Y. (2004). Tumor blood vessels formation in osteosarcoma: Vasculogenesis mimicry. Chin. Med. J..

[253] Sun B., Zhang S., Zhao X., Zhang W., Hao X. (2004). Vasculogenic mimicry is associated with poor survival in patients with mesothelial sarcomas and alveolar rhabdomyosarcomas. Int. J. Oncol..

[254] Ria R., Reale A., De Luisi A., Ferrucci A., Moschetta M., Vacca A. (2011). Bone marrow angiogenesis and progression in multiple myeloma. Am. J. Blood Res..

[255] Vacca A., Ria R., Reale A., Ribatti D. (2014). Angiogenesis in multiple myeloma. Chem. Immunol. Allergy.

[256] Ellis L.M., Fidler I.J. (2010). Finding the tumor copycat. Therapy fails, patients don’t. Nat. Med..

[257] Pinto M.P., Sotomayor P., Carrasco-Avino G., Corvalan A.H., Owen G.I. (2016). Escaping Antiangiogenic Therapy: Strategies Employed by Cancer Cells. Int. J. Mol. Sci..

[258] Huijbers E.J., van Beijnum J.R., Thijssen V.L., Sabrkhany S., Nowak-Sliwinska P., Griffioen A.W. (2016). Role of the tumor stroma in resistance to anti-angiogenic therapy. Drug Resist. Updat.

[259] Kotyza J. (2017). Chemokines in tumor proximal fluids. Biomed. Pap. Med. Fac. Univ. Palacky Olomouc. Czech Repub..

[260] Pries R., Wollenberg B. (2006). Cytokines in head and neck cancer. Cytokine Growth Factor Rev..

[261] Cao Y. (2016). Future options of anti-angiogenic cancer therapy. Chin. J. Cancer.

[262] Rytlewski J.A., Alejandra Aldon M., Lewis E.W., Suggs L.J. (2015). Mechanisms of tubulogenesis and endothelial phenotype expression by MSCs. Microvasc. Res..

[263] Cheng L., Huang Z., Zhou W., Wu Q., Donnola S., Liu J.K., Fang X., Sloan A.E., Mao Y., Lathia J.D. (2013). Glioblastoma stem cells generate vascular pericytes to support vessel function and tumor growth. Cell.

[264] Shenoy A.K., Jin Y., Luo H.C., Tang M., Pampo C., Shao R., Siemann D.W., Wu L.Z., Heldermon C.D., Law B.K. (2016). Epithelial-to-mesenchymal transition confers pericyte properties on cancer cells. J. Clin. Investig..

[265] Braeuer R.R., Watson I.R., Wu C.J., Mobley A.K., Kamiya T., Shoshan E., Bar-Eli M. (2014). Why is melanoma so metastatic?. Pigm. Cell Melanoma Res..

[266] Chen J.A., Shi M., Li J.Q., Qian C.N. (2010). Angiogenesis: Multiple masks in hepatocellular carcinoma and liver regeneration. Hepatol. Int..

[267] Timar J., Tovari J., Raso E., Meszaros L., Bereczky B., Lapis K. (2005). Platelet-mimicry of cancer cells: Epiphenomenon with clinical significance. Oncology.

[268] Kotiyal S., Bhattacharya S. (2015). Epithelial Mesenchymal Transition and Vascular Mimicry in Breast Cancer Stem Cells. Crit. Rev. Eukaryot. Gene Expr..

[269] Lin X., Sun B.C., Zhu D.W., Zhao X.L., Sun R., Zhang Y.H., Zhang D.F., Dong X.Y., Gu Q., Li Y.L. (2016). Notch4+cancer stem-like cells promote the metastatic and invasive ability of melanoma. Cancer Sci..

[270] Guo X., Xu S., Gao X., Wang J., Xue H., Chen Z., Zhang J., Guo X., Qian M., Qiu W. (2017). Macrophage migration inhibitory factor promotes vasculogenic mimicry formation induced by hypoxia via CXCR4/AKT/EMT pathway in human glioblastoma cells. Oncotarget.

[271] Priya S.K., Nagare R.P., Sneha V.S., Sidhanth C., Bindhya S., Manasa P., Ganesan T.S. (2016). Tumour angiogenesis-Origin of blood vessels. Int. J. Cancer.

[272] Burrell K., Zadeh G., Ran S. (2012). Molecular Mechanisms of Tumor Angiogenesis. Tumor Angiogenesis.

[273] Shahneh F.Z., Baradaran B., Zamani F., Aghebati-Maleki L. (2013). Tumor angiogenesis and anti-angiogenic therapies. Hum. Antibodies.

[274] Plate K.H., Scholz A., Dumont D.J. (2012). Tumor angiogenesis and anti-angiogenic therapy in malignant gliomas revisited. Acta Neuropathol..

[275] Paulis Y.W.J., Soetekouw P.M., Verheul H.M., Tjan-Heijnen V.C., Griffioen A.W. (2010). Signalling pathways in vasculogenic mimicry. Biochim. Biophys. Acta.

[276] Delgado-Bellido D., Serrano-Saenz S., Fernandez-Cortes M., Oliver F.J. (2017). Vasculogenic mimicry signaling revisited: Focus on non-vascular VE-cadherin. Mol. Cancer.

[277] Seftor E.A., Meltzer P.S., Schatteman G.C., Gruman L.M., Hess A.R., Kirschmann D.A., Seftor R.E., Hendrix M.J. (2002). Expression of multiple molecular phenotypes by aggressive melanoma tumor cells: Role in vasculogenic mimicry. Crit. Rev. Oncol. Hematol..

[278] Li S., Meng W., Guan Z., Guo Y., Han X. (2016). The hypoxia-related signaling pathways of vasculogenic mimicry in tumor treatment. Biomed. Pharmacother..

[279] Macklin P.S., McAuliffe J., Pugh C.W., Yamamoto A. (2017). Hypoxia and HIF pathway in cancer and the placenta. Placenta.

[280] Bordeleau F., Mason B.N., Lollis E.M., Mazzola M., Zanotelli M.R., Somasegar S., Califano J.P., Montague C., LaValley D.J., Huynh J. (2017). Matrix stiffening promotes a tumor vasculature phenotype. Proc. Natl. Acad. Sci. USA.

[281] Krock B.L., Skuli N., Simon M.C. (2011). Hypoxia-induced angiogenesis: Good and evil. Genes Cancer.

[282] Yang J., Zhu D.M., Zhou X.G., Yin N., Zhang Y., Zhang Z.X., Li D.C., Zhou J. (2017). HIF-2alpha promotes the formation of vasculogenic mimicry in pancreatic cancer by regulating the binding of Twist1 to the VE-cadherin promoter. Oncotarget.

[283] Angara K., Rashid M.H., Shankar A., Ara R., Iskander A., Borin T.F., Jain M., Achyut B.R., Arbab A.S. (2017). Vascular mimicry in glioblastoma following anti-angiogenic and anti-20-HETE therapies. Histol. Histopathol..

[284] Pezzolo A., Marimpietri D., Raffaghello L., Cocco C., Pistorio A., Gambini C., Cilli M., Horenstein A., Malavasi F., Pistoia V. (2014). Failure of anti tumor-derived endothelial cell immunotherapy depends on augmentation of tumor hypoxia. Oncotarget.

[285] Fernandez-Barral A., Orgaz J.L., Gomez V., del Peso L., Calzada M.J., Jimenez B. (2012). Hypoxia negatively regulates antimetastatic PEDF in melanoma cells by a hypoxia inducible factor-independent, autophagy dependent mechanism. PLoS ONE.

[286] Yang J., Zhang X., Zhang Y., Zhu D., Zhang L., Li Y., Zhu Y., Li D., Zhou J. (2016). HIF-2alpha promotes epithelial-mesenchymal transition through regulating Twist2 binding to the promoter of E-cadherin in pancreatic cancer. J. Exp. Clin. Cancer Res..

[287] Alameddine R.S., Hamieh L., Shamseddine A. (2014). From sprouting angiogenesis to erythrocytes generation by cancer stem cells: Evolving concepts in tumor microcirculation. BioMed Res. Int..

[288] Zhang D., Yang X., Yang Z., Fei F., Li S., Qu J., Zhang M., Li Y., Zhang X., Zhang S. (2017). Daughter Cells and Erythroid Cells Budding from PGCCs and Their Clinicopathological Significances in Colorectal Cancer. J. Cancer.

[289] Zhang S., Fu Z., Wei J., Guo J., Liu M., Du K. (2015). Peroxiredoxin 2 is involved in vasculogenic mimicry formation by targeting VEGFR2 activation in colorectal cancer. Med. Oncol..

[290] Dong J., Zhao Y., Huang Q., Fei X., Diao Y., Shen Y., Xiao H., Zhang T., Lan Q., Gu X. (2011). Glioma stem/progenitor cells contribute to neovascularization via transdifferentiation. Stem Cell Rev..

[291] Ren H., Du P., Ge Z., Jin Y., Ding D., Liu X., Zou Q. (2016). TWIST1 and BMI1 in Cancer Metastasis and Chemoresistance. J. Cancer.

[292] Liu K., Sun B., Zhao X., Wang X., Li Y., Qiu Z., Liu T., Gu Q., Dong X., Zhang Y. (2015). Hypoxia promotes vasculogenic mimicry formation by the Twist1-Bmi1 connection in hepatocellular carcinoma. Int. J. Mol. Med..

[293] Sun T., Sun B.C., Zhao X.L., Zhao N., Dong X.Y., Che N., Yao Z., Ma Y.M., Gu Q., Zong W.K. (2011). Promotion of tumor cell metastasis and vasculogenic mimicry by way of transcription coactivation by Bcl-2 and Twist1: A study of hepatocellular carcinoma. Hepatology.

[294] Liang Y., Hu J., Li J., Liu Y., Yu J., Zhuang X., Mu L., Kong X., Hong D., Yang Q. (2015). Epigenetic Activation of TWIST1 by MTDH Promotes Cancer Stem-like Cell Traits in Breast Cancer. Cancer Res..

[295] Li G., Yang Y., Xu S., Ma L., He M., Zhang Z. (2015). Slug signaling is up-regulated by CCL21/CCR7 [corrected] to induce EMT in human chondrosarcoma. Med. Oncol..

[296] Yang Z., Sun B., Li Y., Zhao X., Zhao X., Gu Q., An J., Dong X., Liu F., Wang Y. (2015). ZEB2 promotes vasculogenic mimicry by TGF-β1 induced epithelial-to-mesenchymal transition in hepatocellular carcinoma. Exp. Mol. Pathol..

[297] Puisieux A., Brabletz T., Caramel J. (2014). Oncogenic roles of EMT-inducing transcription factors. Nat. Cell Biol..

[298] Wang H., Lin H., Pan J., Mo C., Zhang F., Huang B., Wang Z., Chen X., Zhuang J., Wang D. (2016). Vasculogenic Mimicry in Prostate Cancer: The Roles of EphA2 and PI3K. J. Cancer.

[299] Hess A.R., Seftor E.A., Seftor R.E.B., Hendrix M.J.C. (2003). Phosphoinositide 3-kinase regulates membrane type 1-matrix metalloproteinase (MMP) and MMP-2 activity during melanoma cell vasculogenic mimicry. Cancer Res..

[300] Zhang J., Qiao L., Liang N., Xie J., Luo H., Deng G., Zhang J. (2016). Vasculogenic mimicry and tumor metastasis. J. BUON.

[301] Robertson G.P. (2007). Mig-7 linked to vasculogenic mimicry. Am. J. Pathol..

[302] Petty A.P., Garman K.L., Winn V.D., Spidel C.M., Lindsey J.S. (2007). Overexpression of carcinoma and embryonic cytotrophoblast cell-specific Mig-7 induces invasion and vessel-like structure formation. Am. J. Pathol..

[303] Hendrix M.J.C., Seftor E.A., Hess A.R., Seftor R.E.B. (2003). Vasculogenic mimicry and tumour-cell plasticity: Lessons from melanoma. Nat. Rev. Cancer.

[304] Petty A.P., Wright S.E., Rewers-Felkins K.A., Yenderrozos M.A., Vorderstrasse B.A., Lindsey J.S. (2009). Targeting Migration inducting gene-7 inhibits carcinoma cell invasion, early primary tumor growth, and stimulates monocyte oncolytic activity. Mol. Cancer Ther..

[305] Sulzmaier F.J., Jean C., Schlaepfer D.D. (2014). FAK in cancer: Mechanistic findings and clinical applications. Nat. Rev. Cancer.

[306] Van den Brule F.A., Buicu C., Baldet M., Sobel M.E., Cooper D.N., Marschal P., Castronovo V. (1995). Galectin-1 modulates human melanoma cell adhesion to laminin. Biochem. Biophys. Res. Commun..

[307] Hsieh S.H., Ying N.W., Wu M.H., Chiang W.F., Hsu C.L., Wong T.Y., Jin Y.T., Hong T.M., Chen Y.L. (2008). Galectin-1, a novel ligand of neuropilin-1, activates VEGFR-2 signaling and modulates the migration of vascular endothelial cells. Oncogene.

[308] Garin M.I., Chu C.C., Golshayan D., Cernuda-Morollon E., Wait R., Lechler R.I. (2007). Galectin-1: A key effector of regulation mediated by CD4+CD25+ T cells. Blood.

[309] Cooper D.N., Massa S.M., Barondes S.H. (1991). Endogenous muscle lectin inhibits myoblast adhesion to laminin. J. Cell Biol..

[310] Mourad-Zeidan A.A., Melnikova V.O., Wang H., Raz A., Bar-Eli M. (2008). Expression profiling of Galectin-3-depleted melanoma cells reveals its major role in melanoma cell plasticity and vasculogenic mimicry. Am. J. Pathol..

[311] Li Y., Sun B., Zhao X., Wang X., Zhang D., Gu Q., Liu T. (2017). MMP-2 and MMP-13 affect vasculogenic mimicry formation in large cell lung cancer. J. Cell. Mol. Med..

[312] Ruffini F., D’Atri S., Lacal P.M. (2013). Neuropilin-1 expression promotes invasiveness of melanoma cells through vascular endothelial growth factor receptor-2-dependent and -independent mechanisms. Int. J. Oncol..

[313] Ruffini F., Levati L., Graziani G., Caporali S., Atzori M.G., D’Atri S., Lacal P.M. (2017). Platelet-derived growth factor-C promotes human melanoma aggressiveness through activation of neuropilin-1. Oncotarget.

[314] Lambrechts D., Lenz H.J., de Haas S., Carmeliet P., Scherer S.J. (2013). Markers of response for the antiangiogenic agent bevacizumab. J. Clin. Oncol..

[315] Graziani G., Lacal P.M. (2015). Neuropilin-1 as Therapeutic Target for Malignant Melanoma. Front. Oncol..

[316] Hardy K.M., Kirschmann D.A., Seftor E.A., Margaryan N.V., Postovit L.M., Strizzi L., Hendrix M.J. (2010). Regulation of the embryonic morphogen Nodal by Notch4 facilitates manifestation of the aggressive melanoma phenotype. Cancer Res..

[317] Jue C., Lin C., Zhisheng Z., Yayun Q., Feng J., Min Z., Haibo W., Youyang S., Hisamitsu T., Shintaro I. (2017). Notch1 promotes vasculogenic mimicry in hepatocellular carcinoma by inducing EMT signaling. Oncotarget.

[318] Zhao N., Sun H., Sun B., Zhu D., Zhao X., Wang Y., Gu Q., Dong X., Liu F., Zhang Y. (2016). miR-27a-3p suppresses tumor metastasis and VM by down-regulating VE-cadherin expression and inhibiting EMT: An essential role for Twist-1 in HCC. Sci. Rep..

[319] Zhao N., Sun B.C., Zhao X.L., Wang Y., Sun H.Z., Dong X.Y., Meng J., Gu Q. (2015). Changes in microRNAs associated with Twist-1 and Bcl-2 overexpression identify signaling pathways. Exp. Mol. Pathol..

[320] Liu W., Lv C., Zhang B., Zhou Q., Cao Z. (2017). MicroRNA-27b functions as a new inhibitor of ovarian cancer-mediated vasculogenic mimicry through suppression of VE-cadherin expression. RNA.

[321] Wu N., Zhao X., Liu M., Liu H., Yao W., Zhang Y., Cao S., Lin X. (2011). Role of microRNA-26b in glioma development and its mediated regulation on EphA2. PLoS ONE.

[322] An L., Liu Y., Wu A., Guan Y. (2013). microRNA-124 inhibits migration and invasion by down-regulating ROCK1 in glioma. PLoS ONE.

[323] Hunt S., Jones A.V., Hinsley E.E., Whawell S.A., Lambert D.W. (2011). MicroRNA-124 suppresses oral squamous cell carcinoma motility by targeting ITGB1. FEBS Lett..

[324] Wang P., Chen L., Zhang J., Chen H., Fan J., Wang K., Luo J., Chen Z., Meng Z., Liu L. (2014). Methylation-mediated silencing of the miR-124 genes facilitates pancreatic cancer progression and metastasis by targeting Rac1. Oncogene.

[325] Liang Y.J., Wang Q.Y., Zhou C.X., Yin Q.Q., He M., Yu X.T., Cao D.X., Chen G.Q., He J.R., Zhao Q. (2013). MiR-124 targets Slug to regulate epithelial-mesenchymal transition and metastasis of breast cancer. Carcinogenesis.

[326] Wan H.Y., Li Q.Q., Zhang Y., Tian W., Li Y.N., Liu M., Li X., Tang H. (2014). MiR-124 represses vasculogenic mimicry and cell motility by targeting amotL1 in cervical cancer cells. Cancer Lett..

[327] Wang Y., Sun B.C., Zhao X.L., Zhao N., Sun R., Zhu D.W., Zhang Y.H., Li Y.L., Gu Q., Dong X.Y. (2016). Twist1-related miR-26b-5p suppresses epithelial-mesenchymal transition, migration and invasion by targeting SMAD1 in hepatocellular carcinoma. Oncotarget.

[328] Wang Y., Sun B., Sun H., Zhao X., Wang X., Zhao N., Zhang Y., Li Y., Gu Q., Liu F. (2016). Regulation of proliferation, angiogenesis and apoptosis in hepatocellular carcinoma by miR-26b-5p. Tumor Biol..

[329] Zhao X., Wang Y., Deng R., Zhang H., Dou J., Yuan H., Hou G., Du Y., Chen Q., Yu J. (2016). miR186 suppresses prostate cancer progression by targeting Twist1. Oncotarget.

[330] Wang Y., Shao N., Mao X.Y., Zhu M.M., Fan W.F., Shen Z.X., Xiao R., Wang C.C., Bao W.P., Xu X.Y. (2016). MiR-4638–5p inhibits castration resistance of prostate cancer through repressing kidins220 expression and PI3K/AKT pathway activity. Oncotarget.

[331] Chen Z., Wang X., Liu R., Chen L., Yi J., Qi B., Shuang Z., Liu M., Li X., Li S. (2017). KDM4B-mediated epigenetic silencing of miRNA-615–5p augments RAB24 to facilitate malignancy of hepatoma cells. Oncotarget.

[332] Gutschner T., Diederichs S. (2012). The hallmarks of cancer: A long non-coding RNA point of view. RNA Biol..

[333] Cao M.X., Jiang Y.P., Tang Y.L., Liang X.H. (2017). The crosstalk between lncRNA and microRNA in cancer metastasis: Orchestrating the epithelial-mesenchymal plasticity. Oncotarget.

[334] Yu C., Xue J., Zhu W., Jiao Y., Zhang S., Cao J. (2015). Warburg meets non-coding RNAs: The emerging role of ncRNA in regulating the glucose metabolism of cancer cells. Tumor Biol..

[335] Beltran-Anaya F.O., Cedro-Tanda A., Hidalgo-Miranda A., Romero-Cordoba S.L. (2016). Insights into the Regulatory Role of Non-coding RNAs in Cancer Metabolism. Front. Physiol..

[336] Li Y., Wu Z., Yuan J., Sun L., Lin L., Huang N., Bin J., Liao Y., Liao W. (2017). Long non-coding RNA MALAT1 promotes gastric cancer tumorigenicity and metastasis by regulating vasculogenic mimicry and angiogenesis. Cancer Lett..

[337] Ruf W., Seftor E.A., Petrovan R.J., Weiss R.M., Gruman L.M., Margaryan N.V., Seftor R.E., Miyagi Y., Hendrix M.J.C. (2003). Differential role of tissue factor pathway inhibitors 1 and 2 in melanoma vasculogenic mimicry. Cancer Res..

[338] Kucera T., Lammert E. (2009). Ancestral vascular tube formation and its adoption by tumors. Biol. Chem..

[339] Qiao L., Liang N., Zhang J., Xie J., Liu F., Xu D., Yu X., Tian Y. (2015). Advanced research on vasculogenic mimicry in cancer. J. Cell. Mol. Med..

[340] Cao Z., Bao M., Miele L., Sarkar F.H., Wang Z., Zhou Q. (2013). Tumour vasculogenic mimicry is associated with poor prognosis of human cancer patients: A systemic review and meta-analysis. Eur. J. Cancer.

[341] Guo Q., Yuan Y., Jin Z., Xu T., Gao Y., Wei H., Li C., Hou W., Hua B. (2016). Association between tumor vasculogenic mimicry and the poor prognosis of gastric cancer in China: An updated systematic review and meta-analysis. BioMed Res. Int..

[342] Liu J., Huang J., Yao W.Y., Ben Q.W., Chen D.F., He X.Y., Li L., Yuan Y.Z. (2012). The origins of vacularization in tumors. Front. Biosci. (Landmark Ed.).

[343] Tan L.Y., Mintoff C., Johan M.Z., Ebert B.W., Fedele C., Zhang Y.F., Szeto P., Sheppard K.E., McArthur G.A., Foster-Smith E. (2016). Desmoglein 2 promotes vasculogenic mimicry in melanoma and is associated with poor clinical outcome. Oncotarget.

[344] Yang J.P., Liao Y.D., Mai D.M., Xie P., Qiang Y.Y., Zheng L.S., Wang M.Y., Mei Y., Meng D.F., Xu L. (2016). Tumor vasculogenic mimicry predicts poor prognosis in cancer patients: A meta-analysis. Angiogenesis.

[345] Zhu B., Zhou L., Yu L., Wu S., Song W., Gong X., Wang D. (2017). Evaluation of the correlation of vasculogenic mimicry, ALDH1, KAI1 and microvessel density in the prediction of metastasis and prognosis in colorectal carcinoma. BMC Surg..

[346] Yu L., Zhu B., Wu S., Zhou L., Song W., Gong X., Wang D. (2017). Evaluation of the correlation of vasculogenic mimicry, ALDH1, KiSS-1, and MACC1 in the prediction of metastasis and prognosis in ovarian carcinoma. Diagn. Pathol..

[347] Yao L., Zhang D., Zhao X., Sun B., Liu Y., Gu Q., Zhang Y., Zhao X., Che N., Zheng Y. (2016). Dickkopf-1-promoted vasculogenic mimicry in non-small cell lung cancer is associated with EMT and development of a cancer stem-like cell phenotype. J. Cell. Mol. Med..

[348] Liu W.B., Xu G.L., Jia W.D., Li J.S., Ma J.L., Chen K., Wang Z.H., Ge Y.S., Ren W.H., Yu J.H. (2011). Prognostic significance and mechanisms of patterned matrix vasculogenic mimicry in hepatocellular carcinoma. Med. Oncol..

[349] Zununi Vahed S., Salehi R., Davaran S., Sharifi S. (2017). Liposome-based drug co-delivery systems in cancer cells. Mater. Sci. Eng. C Mater. Biol. Appl..

[350] Ferrara N., Gerber H.P., LeCouter J. (2003). The biology of VEGF and its receptors. Nat. Med..

[351] Vredenburgh J.J., Desjardins A., Herndon J.E., Dowell J.M., Reardon D.A., Quinn J.A., Rich J.N., Sathornsumetee S., Gururangan S., Wagner M. (2007). Phase II trial of bevacizumab and irinotecan in recurrent malignant glioma. Clin. Cancer Res..

[352] Van der Veldt A.A., Lubberink M., Bahce I., Walraven M., de Boer M.P., Greuter H.N., Hendrikse N.H., Eriksson J., Windhorst A.D., Postmus P.E. (2012). Rapid decrease in delivery of chemotherapy to tumors after anti-VEGF therapy: Implications for scheduling of anti-angiogenic drugs. Cancer Cell.

[353] Siemann D.W. (2011). The unique characteristics of tumor vasculature and preclinical evidence for its selective disruption by tumor-vascular disrupting agents. Cancer Treat. Rev..

[354] Lin Z., Zhang Q., Luo W. (2016). Angiogenesis inhibitors as therapeutic agents in cancer: Challenges and future directions. Eur. J. Pharmacol..

[355] Mahase S., Rattenni R.N., Wesseling P., Leenders W., Baldotto C., Jain R., Zagzag D. (2017). Hypoxia-mediated mechanisms associated with antiangiogenic treatment resistance in glioblastomas. Am. J. Pathol..

[356] Pezzella F., Gatter K., Qian C.N. (2016). Twenty years after: The beautiful hypothesis and the ugly facts. Chin. J. Cancer.

[357] Jain R.K. (2014). Antiangiogenesis strategies revisited: From starving tumors to alleviating hypoxia. Cancer Cell.

[358] Folkman J. (1995). Angiogenesis in cancer, vascular, rheumatoid and other disease. Nat. Med..

[359] Boehm T., Folkman J., Browder T., O’Reilly M.S. (1997). Antiangiogenic therapy of experimental cancer does not induce acquired drug resistance. Nature.

[360] Ribatti D. (2010). The inefficacy of antiangiogenic therapies. J. Angiogenes Res..

[361] Sennino B., McDonald D.M. (2012). Controlling escape from angiogenesis inhibitors. Nat. Rev. Cancer.

[362] De Falco S. (2014). Antiangiogenesis therapy: An update after the first decade. Korean J. Intern. Med..

[363] Jayson G.C., Kerbel R., Ellis L.M., Harris A.L. (2016). Antiangiogenic therapy in oncology: Current status and future directions. Lancet.

[364] Crawford Y., Ferrara N. (2009). Tumor and stromal pathways mediating refractoriness/resistance to anti-angiogenic therapies. Trends Pharmacol. Sci..

[365] Francia G., Emmenegger U., Kerbel R.S. (2009). Tumor-associated fibroblasts as “Trojan Horse” mediators of resistance to anti-VEGF therapy. Cancer Cell.

[366] Hendrix M.J., Seftor E.A., Seftor R.E., Chao J.T., Chien D.S., Chu Y.W. (2016). Tumor cell vascular mimicry: Novel targeting opportunity in melanoma. Pharmacol. Ther..

[367] Seftor R.E., Hess A.R., Seftor E.A., Kirschmann D.A., Hardy K.M., Margaryan N.V., Hendrix M.J. (2012). Tumor cell vasculogenic mimicry: From controversy to therapeutic promise. Am. J. Pathol..

[368] Chung H.J., Mahalingam M. (2014). Angiogenesis, vasculogenic mimicry and vascular invasion in cutaneous malignant melanoma—Implications for therapeutic strategies and targeted therapies. Expert Rev. Anticancer Ther..

[369] Mao J.M., Liu J., Guo G., Mao X.G., Li C.X. (2015). Glioblastoma vasculogenic mimicry: Signaling pathways progression and potential anti-angiogenesis targets. Biomark. Res..

[370] Sasanelli F., Hocking A., Pulford E., Irani Y., Klebe S. (2017). Vasculogenic mimicry in vitro in tumour cells derived from metastatic malignant pleural effusions. Pathology.

[371] Racordon D., Valdivia A., Mingo G., Erices R., Aravena R., Santoro F., Bravo M.L., Ramirez C., Gonzalez P., Sandoval A. (2017). Structural and functional identification of vasculogenic mimicry in vitro. Sci. Rep..

[372] Xu M., Zhu C.H., Zhao X., Chen C., Zhang H.L., Yuan H.H., Deng R., Dou J.Z., Wang Y.L., Huang J. (2015). Atypical ubiquitin E3 ligase complex Skp1-Pam-Fbxo45 controls the core epithelial-to-mesenchymal transition-inducing transcription factors. Oncotarget.

[373] Bianchi M.E., Agresti A. (2005). HMG proteins: Dynamic players in gene regulation and differentiation. Curr. Opin. Genet. Dev..

[374] Lotze M.T., DeMarco R.A. (2003). Dealing with death: HMGB1 as a novel target for cancer therapy. Curr. Opin. Investig. Drugs.

[375] Yin H., Shao Y., Chen X. (2017). The effects of CD147 on the cell proliferation, apoptosis, invasion, and angiogenesis in glioma. Neurol. Sci..

[376] Schultz N.A., Johansen J.S. (2010). YKL-40-A Protein in the Field of Translational Medicine: A Role as a Biomarker in Cancer Patients?. Cancers (Basel).

[377] Shao R., Taylor S.L., Oh D.S., Schwartz L.M. (2015). Vascular heterogeneity and targeting: The role of YKL-40 in glioblastoma vascularization. Oncotarget.

[378] Shao R., Francescone R., Ngernyuang N., Bentley B., Taylor S.L., Moral L., Yan W. (2014). Anti-YKL-40 antibody and ionizing irradiation synergistically inhibit tumor vascularization and malignancy in glioblastoma. Carcinogenesis.

[379] Ribatti D. (2016). Tumor refractoriness to anti-VEGF therapy. Oncotarget.

[380] Chen X., Zhang H., Zhu H., Yang X., Yang Y., Yang Y., Min H., Chen G., Liu J., Lu J. (2016). Endostatin combined with radiotherapy suppresses vasculogenic mimicry formation through inhibition of epithelial-mesenchymal transition in esophageal cancer. Tumor Biol..

[381] Miyata N., Taniguchi K., Seki T., Ishimoto T., Sato-Watanabe M., Yasuda Y., Doi M., Kametani S., Tomishima Y., Ueki T. (2001). HET0016, a potent and selective inhibitor of 20-HETE synthesizing enzyme. Br. J. Pharmacol..

[382] Lv H., Li Y., Du H., Fang J., Song X., Zhang J. (2013). The synthetic compound norcantharidiniInduced apoptosis in mantle cell lymphoma in vivo and in vitro through the PI3K-Akt-NF- kappa B signaling pathway. Evid. Based Complement. Alternat. Med..

[383] Yeh C.B., Hsieh M.J., Hsieh Y.H., Chien M.H., Chiou H.L., Yang S.F. (2012). Antimetastatic effects of norcantharidin on hepatocellular carcinoma by transcriptional inhibition of MMP-9 through modulation of NF-kB activity. PLoS ONE.

[384] Yeh C.B., Hsieh M.J., Hsieh Y.H., Chien M.H., Chiou H.L., Yang S.F. (2017). Correction: Antimetastatic effects of norcantharidin on hepatocellular carcinoma by transcriptional inhibition of MMP-9 through modulation of NF-kB activity. PLoS ONE.

[385] Wang Z., You D., Lu M., He Y., Yan S. (2017). Inhibitory effect of norcantharidin on melanoma tumor growth and vasculogenic mimicry by suppressing MMP-2 expression. Oncol. Lett..

[386] Zhu W., Sun W., Zhang J.T., Liu Z.Y., Li X.P., Fan Y.Z. (2015). Norcantharidin enhances TIMP-2 anti-vasculogenic mimicry activity for human gallbladder cancers through downregulating MMP-2 and MT1-MMP. Int. J. Oncol..

[387] Zhang S., Li M., Gu Y., Liu Z., Xu S., Cui Y., Sun B. (2008). Thalidomide influences growth and vasculogenic mimicry channel formation in melanoma. J. Exp. Clin. Cancer Res..

[388] Guan Y.Y., Luan X., Lu Q., Liu Y.R., Sun P., Zhao M., Chen H.Z., Fang C. (2016). Natural products with antiangiogenic and antivasculogenic mimicry activity. Mini. Rev. Med. Chem..

[389] Su S.J., Yeh T.M., Chuang W.J., Ho C.L., Chang K.L., Cheng H.L., Liu H.S., Cheng H.L., Hsu P.Y., Chow N.H. (2005). The novel targets for anti-angiogenesis of genistein on human cancer cells. Biochem. Pharmacol..

[390] Cong R., Sun Q., Yang L., Gu H., Zeng Y., Wang B. (2009). Effect of Genistein on vasculogenic mimicry formation by human uveal melanoma cells. J. Exp. Clin. Cancer Res..

[391] Liu R., Cao Z., Pan Y., Zhang G., Yang P., Guo P., Zhou Q. (2013). Jatrorrhizine hydrochloride inhibits the proliferation and neovascularization of C8161 metastatic melanoma cells. Anticancer Drugs.

[392] Chen L.X., He Y.J., Zhao S.Z., Wu J.G., Wang J.T., Zhu L.M., Lin T.T., Sun B.C., Li X.R. (2011). Inhibition of tumor growth and vasculogenic mimicry by curcumin through down-regulation of the EphA2/PI3K/MMP pathway in a murine choroidal melanoma model. Cancer Biol. Ther..

[393] Vartanian A.A., Burova O.S., Stepanova E.V., Baryshnikov A.Y., Lichinitser M.R. (2007). Melanoma vasculogenic mimicry is strongly related to reactive oxygen species level. Melanoma Res..

[394] Zang M., Hu L., Zhang B., Zhu Z., Li J., Zhu Z., Yan M., Liu B. (2017). Luteolin suppresses angiogenesis and vasculogenic mimicry formation through inhibiting Notch1-VEGF signaling in gastric cancer. Biochem. Biophys. Res. Commun..

[395] Jue C., Min Z., Zhisheng Z., Lin C., Yayun Q., Xuanyi W., Feng J., Haibo W., Youyang S., Tadashi H. (2017). COE inhibits vasculogenic mimicry in hepatocellular carcinoma via suppressing Notch1 signaling. J. Ethnopharmacol..

[396] Yao N., Ren K., Wang Y., Jin Q., Lu X., Lu Y., Jiang C., Zhang D., Lu J., Wang C. (2017). Paris polyphylla suppresses proliferation and vsculogenic mimicry of human osteosarcoma cells and inhibits tumor growth in vivo. Am. J. Chin. Med..

[397] Yamakawa S., Asai T., Uchida T., Matsukawa M., Akizawa T., Oku N. (2004). (−)-Epigallocatechin gallate inhibits membrane-type 1 matrix metalloproteinase, MT1-MMP, and tumor angiogenesis. Cancer Lett..

[398] Ying M., Chen G., Lu W. (2015). Recent advances and strategies in tumor vasculature targeted nano-drug delivery systems. Curr. Pharm. Des..

[399] Li X.Y., Zhao Y., Sun M.G., Shi J.F., Ju R.J., Zhang C.X., Li X.T., Zhao W.Y., Mu L.M., Zeng F. (2014). Multifunctional liposomes loaded with paclitaxel and artemether for treatment of invasive brain glioma. Biomaterials.

[400] Liu Y., Wu X., Gao Y., Zhang J., Zhang D., Gu S., Zhu G., Liu G., Li X. (2016). Aptamer-functionalized peptide H3CR5C as a novel nanovehicle for codelivery of fasudil and miRNA-195 targeting hepatocellular carcinoma. Int. J. Nanomed..

[401] Ohuchida K., Mizumoto K., Murakami M., Qian L.W., Sato N., Nagai E., Matsumoto K., Nakamura T., Tanaka M. (2004). Radiation to stromal fibroblasts increases invasiveness of pancreatic cancer cells through tumor-stromal interactions. Cancer Res..

[402] Li G., Satyamoorthy K., Herlyn M. (2001). N-cadherin-mediated intercellular interactions promote survival and migration of melanoma cells. Cancer Res..

